# An Evidence-Level Framework for Evaluating Enzyme-Mediated Plastic and Microplastic Transformation

**DOI:** 10.3390/microorganisms14081787

**Published:** 2026-08-14

**Authors:** Luís Felipe Oliva dos Santos, Samanta Shiraishi Kagueyama, Isadora de Brito Hilário, Amanda Rubia de Figueiredo Trindade, José Rivaldo dos Santos Filho, Rosely Aparecida Peralta, Regina de Fátima Peralta Muniz Moreira, Cristina Giatti Marques de Souza, Rita de Cássia Garcia Simão, Adelar Bracht, Rosane Marina Peralta

**Affiliations:** 1 Postgraduate Program in Environmental Biotechnology, State University of Maringá, Maringá 87020-900, PR, Brazil; luisfelipe.oliva11@gmail.com; 2 Postgraduate Program in Food Science, State University of Maringá, Maringá 87020-900, PR, Brazil; samanta.ssk03@gmail.com (S.S.K.); amandafig@outlook.com.br (A.R.d.F.T.); 3 Postgraduate Program in Biochemistry, Department of Biochemistry, State University of Maringá, Maringá 87020-900, PR, Brazil; pg405793@uem.br (I.d.B.H.); joserivaldosf01@gmail.com (J.R.d.S.F.); cgmsouza@uem.br (C.G.M.d.S.); abracht@uem.br (A.B.); 4 Postgraduate Program in Chemistry, Department of Chemistry, Federal University of Santa Catarina, Florianópolis 88040-900, SC, Brazil; rosely.peralta@ufsc.br; 5 Postgraduate Program in Chemical Engineering, Department of Chemical and Food Engineering, Federal University of Santa Catarina, Florianópolis 88040-900, SC, Brazil; regina.moreira@ufsc.br; 6 Laboratory of Molecular Biochemistry, Laboratory of Enzymology and Fermentation Technology, Center for Medical and Pharmaceutical Sciences, State University of Western Paraná (Unioeste), Cascavel 85819-110, PR, Brazil; rita.simao@unioeste.br

**Keywords:** microplastics, plastic transformation, microbial enzymes, evidence-level framework, polymer depolymerization, PETase, laccase, microbial assimilation, carbon tracking, analytical validation

## Abstract

Microbial enzymes have attracted considerable attention as biocatalysts for plastic transformation, yet the experimental evidence supporting reported biodegradation varies substantially in quality and interpretation. Surface-sensitive techniques, molecular-weight analyses, identification of transformation products, microbial assimilation assays, and carbon-tracking approaches each validate different stages of polymer transformation, but they are often treated as equivalent evidence of biodegradation. This review critically examines the analytical basis of enzyme-mediated plastic and microplastic transformation and introduces the Evidence-Level Framework (ELF), which classifies studies according to the highest experimentally validated transformation endpoint, from microbial colonization (ELF 0) to polymer-derived carbon conversion (ELF 5). Systematic screening identified 102 eligible experimental studies from an initial dataset of 150 publications, all of which were classified using the ELF to provide a comprehensive assessment of the current evidence landscape. Most studies clustered within intermediate evidence levels (ELF 2–3), where analytical validation was limited to polymer chain scission or the detection of soluble transformation products. By contrast, only a small proportion demonstrated microbial assimilation or unequivocal polymer-derived carbon conversion. Hydrolysable polyesters and their associated hydrolytic enzymes consistently reached the highest ELF categories because their chemical structure, enzymatic accessibility, and analytical tractability facilitate validation of successive transformation stages. Conventional plastics, however, remain constrained by polymer recalcitrance, limited substrate accessibility, microbial metabolic capacity, and the scarcity of analytical approaches capable of tracking polymer-derived carbon through biological systems. By providing a common framework for interpreting transformation claims, the ELF establishes objective criteria for experimental design, analytical validation, and comparison across independent studies, offering a stronger foundation for more reproducible, mechanistically robust, and environmentally relevant research on microbial plastic transformation.

## 1. Introduction

Microplastics have become pervasive environmental contaminants owing to their widespread distribution, long-term persistence, and continuous generation through the fragmentation of larger plastic materials. They have been detected in marine, freshwater, terrestrial, and atmospheric environments, throughout food webs, and even in human tissues, raising growing concerns about their ecological consequences and potential implications for human health [1,2,3,4,5,6]. Beyond their persistence, microplastics provide substrates for microbial colonization while adsorbing metals, pesticides, hydrocarbons, pharmaceuticals, and numerous other contaminants. These processes create complex microenvironments that shape microbial activity, enzyme function, and polymer transformation at the plastic interface [7].

Microbial enzymes have emerged as one of the most promising biological strategies for mitigating plastic pollution because of their catalytic specificity, environmental compatibility, and potential application in sustainable plastic transformation processes [8,9,10,11]. Experimental studies have documented microbial colonization of plastic surfaces, biofilm development within the plastisphere, and enzyme-mediated modification of a wide variety of synthetic polymers under laboratory conditions [12,13,14,15]. Together, these advances have expanded current understanding of microbial–polymer interactions while accelerating the discovery of novel plastic-transforming enzymes and microbial systems with potential applications in plastic biodegradation.

Despite this rapid progress, a fundamental challenge remains. Evidence supporting enzyme-mediated plastic transformation is generated through highly heterogeneous analytical approaches that range from observations of surface modification to direct measurements of polymer-derived carbon conversion. As a result, microbial colonization, physicochemical aging, surface modification, depolymerization, transformation-product formation, microbial assimilation, and mineralization are often grouped under the broad concept of biodegradation, although they represent distinct biological processes supported by markedly different levels of analytical evidence [16,17,18]. This inconsistency complicates comparisons among studies and frequently leads to overinterpretation of transformation outcomes.

Changes in surface morphology, functional-group composition, or hydrophobicity detected by scanning electron microscopy (SEM), Fourier transform infrared spectroscopy (FTIR), Raman spectroscopy (RS), and related techniques provide valuable evidence of physicochemical modification. On their own, however, these observations cannot demonstrate polymer depolymerization, microbial assimilation, or mineralization of polymer-derived carbon [9,17,19]. Likewise, the detection of soluble oligomers or monomers is insufficient to establish biodegradation unless their subsequent biological utilization is also demonstrated [20,21,22]. Recent reviews have emphasized the need for more rigorous analytical strategies, carbon-resolved validation, and greater methodological consistency in studies of enzyme-mediated plastic transformation [23,24,25]. Yet the field still lacks a broadly adopted evidence-based framework capable of integrating enzymatic mechanisms, analytical methodologies, and progressively validated transformation endpoints into a coherent system for interpreting experimental evidence [17,18]. A recent critical analysis [26] reached a similar conclusion, underscoring the need for more rigorous standards of evidence.

Against this background, this review critically examines microbial enzymes involved in plastic and microplastic transformation from a unified mechanistic and analytical perspective. Rather than focusing primarily on enzyme classes, microbial taxa, degradation mechanisms, or polymer-specific case studies, it introduces the Evidence-Level Framework (ELF) as a standardized approach for classifying enzyme-mediated plastic transformation according to the highest experimentally validated transformation endpoint. The framework was systematically applied to a comprehensive dataset of eligible experimental studies identified through a structured literature search, providing the first evidence-based classification of the literature according to progressively validated stages of polymer transformation. The review also examines the principal classes of plastic-transforming enzymes, the influence of polymer chemistry and environmental conditions on transformation efficiency, and the biological, physicochemical, and analytical factors that currently limit progression toward higher evidence levels. By combining mechanistic insight with analytical validation, the ELF establishes objective criteria for interpreting transformation claims, facilitates comparisons across independent studies, and provides a practical framework for designing more reproducible, mechanistically rigorous, and environmentally relevant research on enzyme-mediated plastic transformation.

## 2. Literature Search, Study Selection, Data Extraction, and Evidence Classification

**Figure 1:**
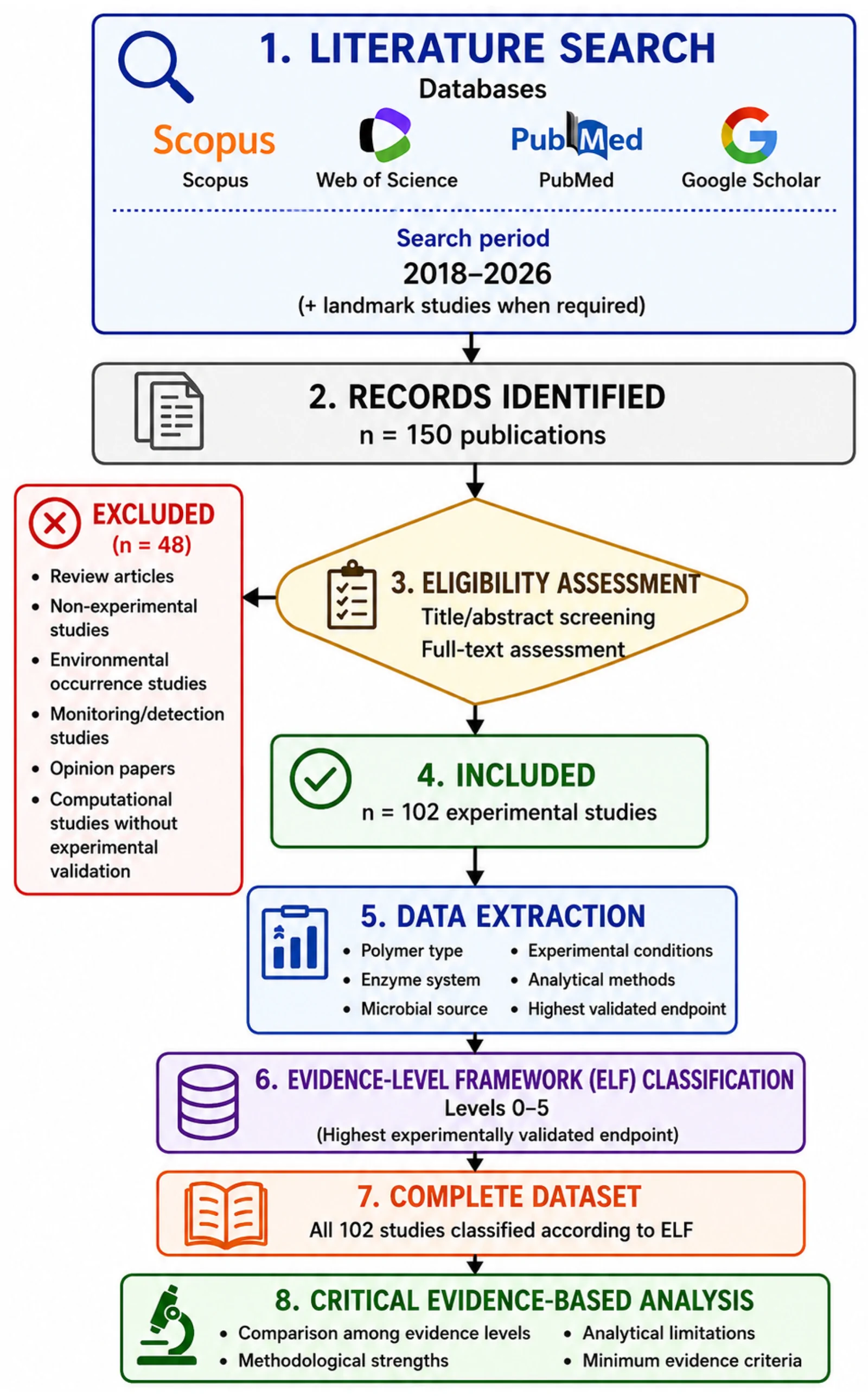
Methodological workflow adopted for literature retrieval, study selection, evidence extraction, and classification using the ELF. After eligibility assessment, 102 original experimental studies were classified into ELF Levels 0–5. The complete dataset is provided in Supplementary Table S1.

A structured literature review was conducted to identify, select, and critically evaluate original experimental studies investigating enzyme-mediated transformation of plastics and microplastics. The methodological workflow adopted for literature retrieval, study selection, data extraction, and classification using the Evidence-Level Framework (ELF) is summarized in Figure 1.

Systematic searches were conducted in Scopus, Web of Science, PubMed, and Google Scholar using database-specific combinations of keywords and Boolean operators. Search terms included plastic biodegradation, microplastic biodegradation, plastic transformation, plastic depolymerization, plastic-degrading enzymes, PETase, cutinase, esterase, lipase, laccase, peroxidase, polyester hydrolase, plastisphere, polymer degradation, polymer assimilation, polymer mineralization, and carbon mineralization, together with related terms where appropriate. The primary search focused on publications published between 2018 and 2026, while earlier landmark studies were retained to provide historical perspective and mechanistic context.

After duplicate removal, titles, abstracts, and full-text articles were screened according to predefined eligibility criteria. Only original experimental studies investigating enzyme-mediated transformation of synthetic plastics or microplastics were considered eligible. Studies describing microbial colonization, surface modification, depolymerization, formation of polymer-derived products, microbial assimilation, or polymer-derived carbon conversion were included only when supported by experimental evidence. Review articles, perspective and opinion papers, conference abstracts, methodological studies lacking experimental validation, computational investigations without experimental confirmation, and studies focused exclusively on the occurrence, monitoring, distribution, or detection of microplastics in environmental matrices or organisms were excluded from evidence classification, although selected publications were retained as background references where appropriate.

Application of these criteria yielded 102 original experimental studies. For each study, information was systematically extracted on polymer type, enzyme class, microbial source, experimental conditions, analytical methodologies, experimentally validated transformation endpoint, principal analytical evidence, and major analytical limitations. The resulting dataset constitutes the complete evidence database generated in this review and is provided in Supplementary Table S1, together with the full bibliographic references for all 102 experimental studies.

All eligible studies were subsequently classified using the Evidence-Level Framework (ELF), developed to organize enzyme-mediated plastic transformation according to progressively validated levels of experimental evidence. The framework was established through comparative evaluation of the analytical methodologies and experimentally validated transformation endpoints reported across the eligible studies. Instead of ranking enzyme systems according to apparent degradation efficiency, the ELF classifies each study according to the highest experimentally validated stage of polymer transformation supported by the available analytical evidence.

**Figure 2:**
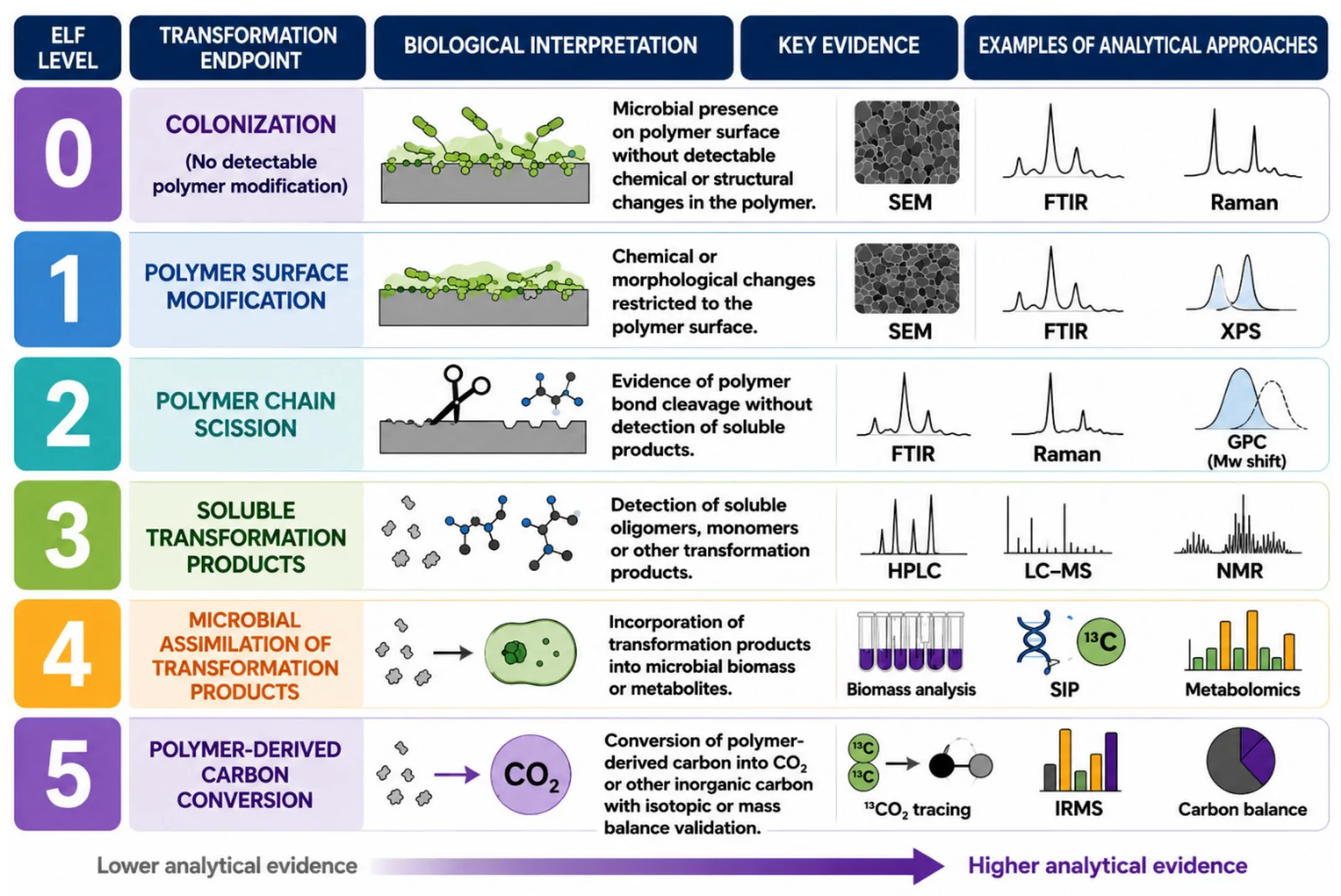
Evidence-Level Framework (ELF) for Microbial-Mediated Plastic Transformation. Conceptual overview of the ELF showing the progression from microbial colonization to polymer-derived carbon conversion, the corresponding transformation endpoints, key analytical evidence, and representative analytical methods.

To ensure methodological consistency, every study was assigned to a single ELF category corresponding to its highest experimentally validated transformation endpoint. Evidence associated with lower categories was considered only when no higher level of experimental validation had been achieved. The conceptual organization of the framework, including the six evidence levels, their corresponding transformation endpoints, and representative analytical methodologies, is presented in Figure 2. The six evidence levels are defined as follows: ELF 0, microbial colonization or biofilm formation without detectable polymer modification; ELF 1, physicochemical surface modification without direct evidence of polymer chain scission; ELF 2, polymer chain scission or molecular-weight reduction without identification of soluble polymer-derived products; ELF 3, identification of soluble oligomers or monomers demonstrating depolymerization without evidence of microbial assimilation; ELF 4, experimental demonstration of microbial assimilation or biological utilization of polymer-derived transformation products; ELF 5, unequivocal polymer-derived carbon conversion demonstrated through isotope tracing, carbon mass-balance approaches, or equivalent carbon-resolved analytical methodologies.

The ELF provides a standardized, evidence-based framework for distinguishing progressively validated stages of enzyme-mediated plastic transformation. By classifying studies according to the strength of their analytical evidence rather than their apparent degradation efficiency, it enables direct comparison across different polymers, enzyme systems, analytical methodologies, and experimental designs.

## 3. Assessing Experimental Evidence for Enzyme-Mediated Plastic Transformation Using the ELF

Research on enzyme-mediated plastic transformation has expanded rapidly over the past decade. Even so, the field still lacks objective criteria for distinguishing fundamentally different levels of experimental evidence. As a result, studies describing microbial colonization, physicochemical surface modification, polymer depolymerization, microbial assimilation, or polymer-derived carbon conversion are often interpreted as providing equivalent evidence of biodegradation, although these observations represent distinct biological processes supported by markedly different analytical methodologies [17,18,19,20,21,22]. This inconsistency complicates comparisons among studies and frequently leads to overinterpretation of transformation outcomes.

The key question is therefore straightforward: how much experimental evidence is required before enzyme-mediated plastic transformation can be considered biologically demonstrated? Answering this question requires distinguishing observations that document early physicochemical interactions at the polymer surface from those demonstrating progressively advanced stages of biological transformation. Surface-sensitive techniques, molecular-weight analyses, identification of soluble transformation products, microbial assimilation assays, and carbon-tracking methodologies each validate different stages of the transformation process. Rather than representing equivalent evidence of biodegradation, these analytical approaches support progressively stronger levels of biological validation [17,18,19,20,21,22].

**Figure 3:**
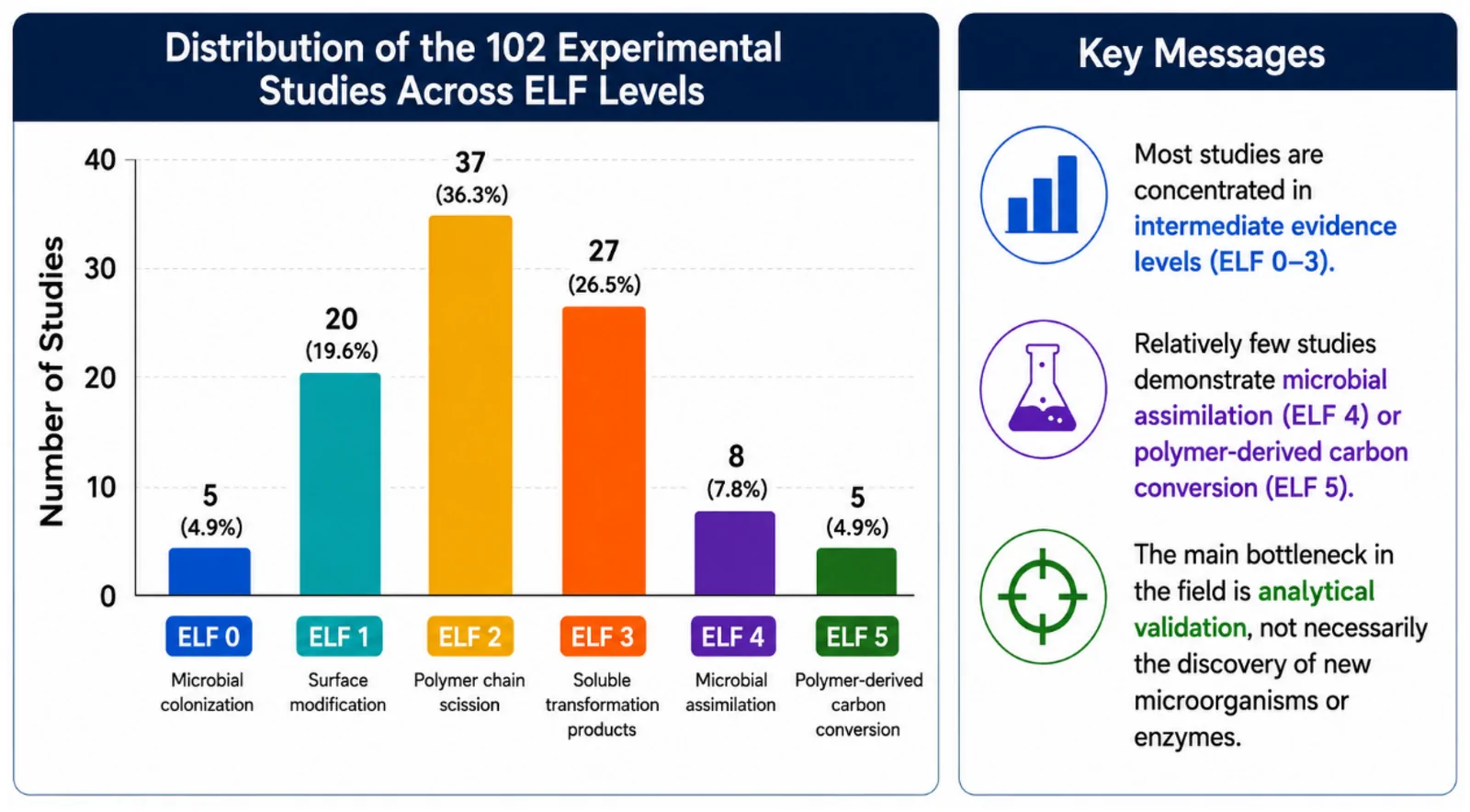
Distribution of the 102 experimental studies across the ELF. The histogram shows the number and percentage of studies classified at each ELF level according to the highest experimentally validated endpoint (ELFs 0–5). The lower panel summarizes the progressive increase in analytical evidence and biological relevance from microbial colonization (ELF 0) to polymer-derived carbon conversion (ELF 5). The distribution highlights that most studies reached intermediate evidence levels (ELFs 2–3), whereas relatively few demonstrated microbial assimilation of polymer-derived carbon (ELF 4) or complete carbon conversion/mineralization (ELF 5). A complete and detailed classification of all studies is provided in Supplementary Table S1.

To examine this progression systematically, the ELF was applied to all 102 eligible experimental studies identified through the methodology described in Section 2. Each study was assigned to the highest evidence level supported by its experimentally validated transformation endpoint. The complete and detailed classification is presented in Supplementary Table S1, while Figure 3 summarizes the distribution of studies across the six ELF categories. Together, they provide an overview of the current evidence landscape, illustrating the transition from microbial colonization (ELF 0) to polymer-derived carbon conversion (ELF 5) and allowing the maturity of the available experimental evidence supporting plastic biodegradation to be assessed immediately.

The distribution shown in Figure 3 indicates that the principal bottleneck in the current literature is no longer the identification of plastic-transforming enzymes, but the implementation of analytical workflows capable of validating microbial assimilation and polymer-derived carbon conversion. Of the 102 experimental studies analyzed, 64 (62.7%) reached intermediate evidence levels (ELFs 2–3), demonstrating polymer chain scission or the formation of soluble transformation products. By contrast, only 13 studies (12.7%) progressed to ELF 4–5 by providing direct evidence of microbial assimilation or polymer-derived carbon conversion. This pronounced imbalance shows that extracellular polymer transformation is now documented in many experimental systems, whereas rigorous validation of the biological fate of polymer-derived carbon remains comparatively rare.

Studies reporting apparently similar transformation outcomes may occupy different ELF categories because the underlying analytical evidence differs substantially. One investigation may document only polymer chain scission, whereas another demonstrates microbial assimilation or polymer-derived carbon conversion using carbon-resolved analytical approaches. The ELF captures this distinction by classifying studies according to the strength of the supporting evidence rather than the apparent extent of polymer transformation [17,18]. Lower ELF categories therefore should not be interpreted as evidence of poor enzymatic performance. Instead, they indicate that experimental validation has not progressed beyond earlier stages of the transformation process. Polymer chemistry, substrate accessibility, microbial physiology, experimental design, and the analytical methodologies selected for validation frequently influence the final ELF category as much as the intrinsic catalytic properties of the enzymes themselves [17,21,22]. Consequently, enzyme systems with comparable catalytic performance may occupy different ELF categories if one study documents only surface modification while another demonstrates microbial assimilation or polymer-derived carbon conversion. Even studies assigned to lower evidence levels can therefore provide valuable mechanistic insights despite lacking sufficient analytical support to validate advanced stages of polymer transformation.

The ELF shifts the interpretation of enzyme-mediated plastic transformation from enzyme performance to evidential strength. Instead of asking whether a particular enzyme appears to degrade a polymer, the framework asks whether the available analytical evidence adequately supports the proposed biological conclusion. This perspective provides a common basis for comparing studies involving different polymers, enzyme systems, analytical methodologies, and experimental designs while reducing inconsistencies arising from the heterogeneous use of the term biodegradation [17,18,21].

Viewed collectively, these findings indicate that the greatest challenge facing enzyme-mediated plastic transformation research is methodological as much as biological. Future advances will depend not only on discovering additional plastic-transforming enzymes, but also on adopting integrated analytical strategies capable of linking extracellular polymer modification with polymer depolymerization, microbial assimilation, metabolic utilization, and unequivocal polymer-derived carbon conversion through carbon-resolved validation [9,17,18,19,20,21,22].

Within this context, the ELF offers a standardized framework for interpreting transformation claims, improving reproducibility and comparability among studies, and establishing objective benchmarks for future investigations. This interpretation is consistent with the critical analysis of Jendrossek [26], who likewise concluded that the principal limitation of the current literature lies not in the observation of polymer surface modification or apparent degradation, but in the insufficient analytical validation required to demonstrate true biodegradation. The following sections build on this perspective by examining why certain enzyme–polymer systems consistently reach higher ELF categories whereas others remain confined to lower evidence levels, emphasizing the combined influence of polymer chemistry, catalytic mechanisms, microbial metabolism, and analytical validation.

## 4. Why Do Some Enzyme Systems Reach Higher Evidence Levels?

### 4.1. Hydrolysable Polyesters as Experimental Benchmarks

Application of the ELF reveals a consistent pattern across the current literature: studies involving hydrolysable polyesters are considerably more likely to reach advanced levels of experimentally validated polymer transformation than those investigating conventional plastics (Supplementary Table S1). This distribution represents one of the clearest trends emerging from the systematic classification of the 102 experimental studies analyzed in this review. It does not imply, however, that hydrolysable polyester is intrinsically easier to biodegrade under environmentally relevant conditions. Instead, they provide experimental systems in which polymer chemistry, enzymatic accessibility, and analytical validation are favorably aligned, allowing successive stages of polymer transformation to be documented with greater confidence [17,21,22].

Unlike polyolefins and other polymers dominated by chemically stable carbon–carbon backbones, hydrolysable polyesters contain ester linkages susceptible to nucleophilic attack. Cleavage of these bonds generates soluble oligomers and monomers that can be directly detected, quantified, and structurally characterized using chromatographic and spectrometric techniques. These characteristics create an experimental setting in which progression from polymer modification to depolymerization, transformation-product formation, microbial assimilation, and, in some cases, polymer-derived carbon conversion can be validated through progressively stronger analytical evidence [9,19,20,21].

Polymer chemistry alone, however, does not account for the higher evidence levels achieved by these materials. Their physicochemical properties are equally important in determining susceptibility to enzymatic transformation. Lower crystallinity increases chain mobility and exposes a greater proportion of amorphous domains, facilitating enzyme access to susceptible bonds. Reduced hydrophobicity and greater molecular flexibility likewise enhance interactions between the polymer surface and the surrounding aqueous environment, improving substrate accessibility and promoting more efficient catalytic attack. Collectively, these properties favor progressive polymer transformation while enabling successive transformation stages to be resolved with complementary analytical methodologies and a high degree of confidence [17].

Poly(lactic acid) (PLA), polyhydroxyalkanoates (PHAs), polybutylene adipate terephthalate (PBAT), and polyethylene terephthalate (PET) illustrate this relationship particularly well. In these systems, soluble intermediates generated during depolymerization can often be monitored throughout the transformation process, making it possible to distinguish polymer chain cleavage from subsequent biological events such as microbial uptake and carbon conversion. Their frequent occurrence in the highest ELF categories therefore reflects the ability to validate each successive stage of transformation with greater analytical confidence, rather than inherently superior biodegradability relative to conventional plastics [13,14,22].

Hydrolysable polyesters have also established many of the methodological standards that now underpin the field. Studies involving these polymers commonly combine molecular-weight analysis, chromatographic identification of transformation products, respirometric measurements, stable isotope labeling, and carbon mass-balance approaches to validate successive stages of polymer transformation. Integrating multiple analytical methodologies provides considerably stronger mechanistic support than reliance on surface-sensitive techniques alone and illustrates the level of experimental rigor required to reach the highest ELF categories [9,18,19,21].

Hydrolysable polyesters should not be regarded as direct environmental analogues of the plastics that dominate global pollution. Conventional materials such as polyethylene, polypropylene, polystyrene, and polyvinyl chloride differ fundamentally in chemical architecture and physicochemical behavior, presenting substantially greater barriers to enzymatic attack [22,27,28,29]. The importance of hydrolysable polyesters lies instead in their role as experimental benchmark systems. They define the mechanistic and analytical standards required to validate advanced stages of polymer transformation while providing a reference against which more recalcitrant polymer systems can be critically evaluated. Their frequent occurrence in the highest ELF categories therefore reflects analytical tractability rather than superior environmental biodegradability. The catalytic mechanisms that make this progression possible are examined in the following subsection.

### 4.2. Hydrolytic Enzymes as Models of Advanced Polymer Transformation

The favorable physicochemical characteristics of hydrolysable polyesters alone are insufficient to explain the higher ELF categories achieved by these systems. Progression from surface modification to depolymerization, transformation-product formation, microbial assimilation, and polymer-derived carbon conversion ultimately depend on enzymes capable of catalyzing polymer backbone cleavage with sufficient efficiency and specificity. Among the enzyme classes investigated to date, hydrolytic enzymes provide the strongest experimental evidence supporting advanced stages of plastic transformation [17,20,29,30]. This trend is clearly reflected in Supplementary Table S1, where hydrolytic enzyme systems account for most studies reaching the highest ELF categories, whereas oxidative enzyme systems remain concentrated within the lower evidence levels. The difference reflects not only distinct catalytic mechanisms but also the greater analytical accessibility of the transformation products generated during polyester hydrolysis.

Hydrolytic enzymes, including PET hydrolases (PETases), cutinases, esterases, and lipases, share a common catalytic principle by hydrolyzing ester bonds within susceptible polymer chains to generate soluble oligomers and monomers that can be detected and quantified using complementary analytical techniques. Unlike oxidative enzymes, whose activity is generally restricted to indirect surface oxidation, hydrolytic enzymes directly reduce polymer molecular weight through backbone cleavage, providing unequivocal molecular evidence of depolymerization [17,29,30]. This direct relationship between catalytic activity and analytically detectable transformation products enables progression through successive ELF categories, from depolymerization to microbial assimilation and, in some experimental systems, polymer-derived carbon conversion.

PETases have become the best-characterized model of enzyme-mediated plastic transformation. Beyond representing the most extensively studied hydrolytic enzymes, they also constitute the principal experimental models for validating progression through the ELF. Since the discovery of the PET-degrading bacterium *Ideonella sakaiensis*, enzymatic hydrolysis of polyethylene terephthalate has been investigated in considerable mechanistic detail. PETases catalyze cleavage of ester bonds within PET, producing soluble intermediates such as mono-(2-hydroxyethyl) terephthalate (MHET), bis-(2-hydroxyethyl) terephthalate (BHET), terephthalic acid (TPA), and ethylene glycol (EG). Because these compounds can be readily identified and quantified by chromatographic and spectrometric methods, PETase-based systems frequently provide robust analytical evidence for ELF Levels 2–4 and, when coupled with microbial metabolism, may ultimately support ELF Level 5 through demonstration of polymer-derived carbon conversion [9,20,29,30,31].

Progress in PET depolymerization has been driven not only by the discovery of new enzymes but also by advances in protein engineering. Rational design, directed evolution, and, more recently, machine learning-guided engineering have substantially improved enzyme thermostability, catalytic efficiency, substrate affinity, and tolerance to industrial operating conditions. Engineered variants such as FAST-PETase and optimized leaf-branch compost cutinases (LCC derivatives) have demonstrated accelerated depolymerization of post-consumer PET under conditions approaching industrial relevance, illustrating how enzyme engineering can overcome important kinetic limitations associated with polymer hydrolysis [29,31,32,33,34,35]. Improved enzyme performance also facilitates progression toward higher ELF categories by increasing both depolymerization efficiency and the production of analytically traceable transformation products.

Cutinases represent a second major group of hydrolytic enzymes contributing to advanced polymer transformation. Originally evolved to hydrolyze the plant cuticle, these enzymes exhibit broad substrate specificity toward synthetic aliphatic and aromatic polyesters, including PET, PBAT, PLA, and related materials. Their relatively high thermostability, catalytic versatility, and compatibility with protein-engineering approaches have made them attractive candidates for enzymatic recycling and polymer valorization. Lipases and esterases may also catalyze polyester hydrolysis, although their performance depends more strongly on polymer accessibility, crystallinity, reaction temperature, and substrate morphology [17,31,32,33,34,35]. Together, these enzyme systems illustrate how catalytic specificity and substrate chemistry jointly influence progression toward higher evidence levels within the ELF.

Efficient depolymerization, however, should not be equated with complete biodegradation. Many studies report substantial release of soluble transformation products without subsequently demonstrating microbial uptake, intracellular metabolism, or conversion into biomass and inorganic carbon. Under these circumstances, even highly efficient hydrolytic enzymes remain confined to intermediate ELF categories because analytical validation ends with depolymerization or product identification. Reaching the highest evidence levels requires integration of extracellular catalysis with microbial metabolic competence and carbon-resolved analytical approaches capable of tracing the biological fate of polymer-derived carbon [18,19,21,22]. Enzyme performance and evidence level should therefore be regarded as related, but fundamentally distinct, concepts within the ELF. Catalytic efficiency determines the capacity to depolymerize the polymer, whereas the ELF reflects the extent to which successive stages of biological transformation have been experimentally validated.

Hydrolytic enzymes currently provide the clearest experimental models of advanced polymer transformation because their catalytic mechanisms generate measurable molecular products that can be linked to successive biological processes. Their frequent progression to higher ELF categories reflects the combined influence of enzyme specificity, polymer chemistry, microbial metabolism, and analytical validation rather than catalytic efficiency alone. They should therefore be regarded primarily as mechanistic and analytical benchmark models that define the level of experimental evidence required to demonstrate advanced polymer transformation. This mechanistic perspective provides the basis for understanding why conventional carbon–carbon backbone polymers rarely achieve comparable evidence levels, a question addressed in the following subsection.

### 4.3. From Experimental Benchmarks to Environmental Reality

The remarkable progress achieved with hydrolysable polyesters and hydrolytic enzymes has transformed current understanding of enzyme-mediated polymer transformation. These systems have established the mechanistic basis of enzymatic depolymerization, driven the development of increasingly efficient biocatalysts, and demonstrated that polymer-derived carbon can, under favorable conditions, enter microbial metabolism. The systematic classification presented in Supplementary Table S1 reflects this trend clearly: the highest ELF categories are dominated by hydrolysable polyester systems despite their comparatively limited environmental representation. These achievements, however, should not be interpreted as representative of the plastics that dominate environmental pollution [17].

Most plastic waste released into terrestrial and aquatic ecosystems consists of conventional polymers such as polyethylene (PE), polypropylene (PP), polystyrene (PS), and polyvinyl chloride (PVC), all of which differ fundamentally from hydrolysable polyesters in both chemical structure and physicochemical behavior. Their chemically stable carbon–carbon backbones, high hydrophobicity, elevated molecular weight, and frequently high crystallinity create substantial barriers to enzyme adsorption, catalytic attack, and polymer-chain cleavage [22,32,36]. These characteristics greatly restrict enzyme accessibility, catalytic efficiency, microbial utilization, and ultimately the progression toward higher ELF categories under environmentally relevant conditions. Consequently, the analytical progression routinely documented for polyester-based systems is only rarely observed for the polymers that predominate in natural environments.

This distinction has important implications for interpreting the current literature. Studies reporting successful depolymerization of PET or other hydrolysable polyesters provide valuable mechanistic insight into what can be achieved under favorable substrate conditions. Directly extrapolating these findings to recalcitrant polyolefins, however, risks creating unrealistic expectations regarding enzyme performance and the feasibility of biological plastic remediation. In many cases, polymer chemistry exerts a greater influence on transformation outcomes than differences among enzyme classes themselves [17]. As a result, the physicochemical properties of the polymer frequently determine the highest ELF category that can realistically be achieved, irrespective of the intrinsic catalytic efficiency of the enzyme employed.

From the perspective of the ELF, hydrolysable polyesters are best regarded as experimental benchmark systems rather than universal models of plastic biodegradation. Their favorable performance reflects the combined influence of polymer chemistry, enzyme accessibility, and analytical tractability rather than the behavior of the plastics that dominate environmental pollution [17,33,37]. Their principal contribution is not to suggest that all plastics can be transformed with comparable efficiency, but to establish the analytical and mechanistic standards required to validate successive stages of polymer transformation. By generating well-defined soluble intermediates that can be tracked throughout the transformation process, these systems define the experimental criteria needed to distinguish surface modification from polymer depolymerization, depolymerization from microbial assimilation, and microbial assimilation from unequivocal polymer-derived carbon conversion [9,18,19,21]. They therefore serve primarily as methodological benchmarks rather than representative models of environmental plastic biodegradation.

Recognizing the distinction between benchmark systems and environmentally relevant polymers also reframes the central question addressed in this review. Instead of asking whether microbial enzymes can transform plastics, a more informative question is why advanced transformation remains comparatively uncommon for the polymers that predominate in natural environments. Conventional plastics, particularly polyolefins, possess chemically stable carbon–carbon backbones, high hydrophobicity, and limited enzymatic accessibility, creating barriers that frequently prevent progression toward higher ELF categories [17,32,33,36]. Explaining this disparity requires consideration of additional physicochemical, microbiological, ecological, and analytical constraints that interrupt successive stages of experimentally validated polymer transformation.

**Figure 4:**
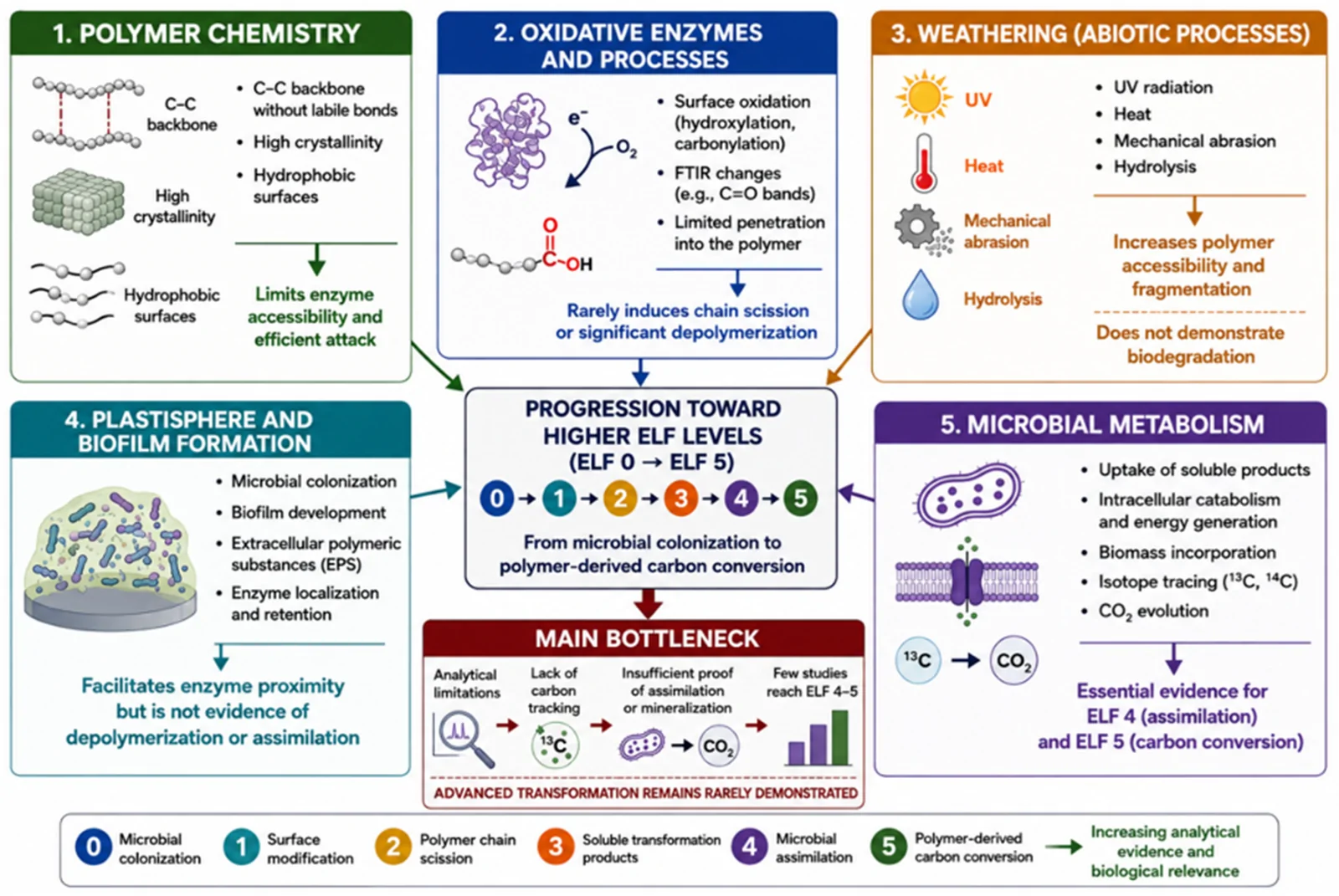
Conceptual overview of the principal factors limiting progression through the Evidence-Level Framework (ELF). The diagram summarizes the interconnected biological, physicochemical, and analytical factors that constrain progression from lower (ELFs 0–2) to higher (ELFs 4–5) evidence levels during enzyme-mediated plastic transformation. Polymer chemistry, oxidative catalytic mechanisms, environmental weathering, plastisphere development, and microbial metabolism collectively influence whether transformation advances beyond surface modification toward polymer depolymerization, microbial assimilation, and polymer-derived carbon conversion. The figure emphasizes that the principal bottleneck in the current literature is not the discovery of new plastic-transforming enzymes, but the integration of analytical approaches capable of unequivocally validating carbon flow from polymers into microbial metabolism.

These interacting constraints are summarized conceptually in Figure 4 and examined in detail in the following section. Together, they show that progression toward higher ELF categories depends on far more than the availability of plastic-transforming enzymes. Polymer chemistry, catalytic mechanisms, environmental weathering, plastisphere development, microbial metabolism, and the analytical methodologies used to validate each transformation stage all contribute to the final level of experimental evidence [8,18]. This integrated perspective provides the conceptual basis for understanding why robust evidence of plastic biodegradation remains comparatively uncommon despite the continuing discovery of new plastic-transforming enzymes.

## 5. Why Do Most Enzyme-Mediated Systems Remain at Lower Evidence Levels?

The benchmark systems discussed in Section 4 define the biological and analytical conditions under which advanced polymer transformation can be experimentally demonstrated. However, application of ELF revealed that most of the 102 experimental studies remain concentrated at the lower and intermediate evidence categories (ELFs 0–3) (Figure 3). This distribution indicates that progression toward microbial assimilation (ELF 4) and polymer-derived carbon conversion (ELF 5) is constrained by multiple interconnected factors rather than by enzyme activity alone [17,18,19,20,21,22].

Progression through the ELF depends on the combined influence of polymer chemistry, enzyme catalytic mechanisms, environmental weathering, plastisphere development, microbial metabolic capacity, and the analytical methodologies used to validate each transformation stage. These biological, physicochemical, and methodological constraints are summarized in Figure 4, which provides the conceptual framework for the following discussion and illustrates why only a limited number of studies currently achieve the highest evidence levels [8,18].

The following subsections examine each of these factors in detail, emphasizing how they influence the transition from initial polymer colonization and surface modification to polymer depolymerization, microbial assimilation, and ultimately polymer-derived carbon conversion.

### 5.1. Oxidative Enzymes Predominantly Support Early Stages of Polymer Transformation

Oxidative enzymes constitute one of the most extensively investigated enzyme groups for the transformation of conventional plastics, particularly polyethylene (PE), polypropylene (PP), polystyrene (PS), polyvinyl chloride (PVC), and other polymers containing chemically stable carbon–carbon backbones. Laccases, manganese peroxidases (MnPs), lignin peroxidases (LiP), versatile peroxidases (VPs), and dye-decolorizing peroxidases (DyPs) have all been reported to promote measurable physicochemical modifications of polymer surfaces through radical-mediated oxidation reactions [17,22,32]. Classification of these studies using the ELF shows that they remain concentrated within Levels 0–2, indicating that surface modification alone seldom progresses to experimentally validated polymer depolymerization (Supplementary Table S1).

Unlike hydrolytic enzymes, oxidative enzymes do not directly cleave polymer backbones through specific hydrolytic reactions. Their catalytic activity relies on the generation of highly reactive radical species that introduce oxygen-containing functional groups, increase surface hydrophilicity, alter surface roughness, and modify polymer physicochemical properties [21,22,32]. These modifications facilitate microbial attachment, biofilm development, and subsequent colonization, representing important early stages of polymer transformation. They do not, however, demonstrate polymer-chain cleavage or the generation of biologically available low-molecular-weight products.

This distinction underlies one of the most common sources of overinterpretation in the literature. Reductions in contact angle, changes in FTIR spectra, increased carbonyl indices, or alterations in surface morphology observed by scanning electron microscopy (SEM) are frequently interpreted as evidence of biodegradation. Jendrossek [26] reached a similar conclusion, arguing that polymer surface oxidation, minor weight loss, or the detection of carbonyl groups cannot be considered evidence of polyethylene biodegradation unless polymer-derived carbon mineralization has been experimentally demonstrated. Although these observations clearly document oxidative modification of the polymer surface, they generally provide evidence only for physicochemical alteration rather than polymer depolymerization [18,21,22]. This explains why many oxidative enzyme systems summarized in Supplementary Table S1 remain confined to the lower ELF categories despite measurable catalytic activity.

The limited progression toward higher evidence levels reflects both enzymatic and polymer-related constraints. Conventional polyolefins possess highly stable carbon–carbon backbones with few chemically accessible functional groups, restricting enzyme binding and limiting catalytic specificity. High crystallinity, hydrophobicity, and low chain mobility further reduce enzyme accessibility, confining oxidative modification largely to the polymer surface [17,32,36]. Under these conditions, oxidative enzymes frequently initiate surface activation without generating sufficient backbone cleavage to support extensive depolymerization or subsequent microbial assimilation.

This apparent limitation should not be interpreted as evidence that oxidative enzymes play only a minor role in plastic transformation. Surface oxidation is likely to represent a critical preparatory step that increases polymer accessibility to subsequent biological or physicochemical processes. By enhancing microbial colonization, promoting enzyme adsorption, facilitating surface weathering, and supporting synergistic interactions with hydrolytic enzymes or microbial consortia, oxidative modification creates conditions that favor later stages of transformation [21,38]. Their principal contribution therefore lies in initiating polymer transformation rather than completing biodegradation.

Viewed within the context of the ELF, oxidative enzymes primarily facilitate the transition from pristine polymer surfaces to biologically accessible interfaces, corresponding predominantly to the lower evidence levels of the framework [17,21,38,39]. Progression beyond these stages requires additional physicochemical, microbiological, and metabolic processes capable of promoting polymer depolymerization, microbial assimilation, and ultimately polymer-derived carbon conversion [18,19,21,22]. The next subsection examines how the intrinsic physicochemical properties of plastics further constrain this progression.

### 5.2. Polymer Chemistry Defines the Upper Limit of Enzyme-Mediated Transformation

The chemical structure of the polymer is one of the primary factors determining whether enzyme-mediated transformation can progress beyond the initial ELF categories. Classification of the representative studies summarized in Supplementary Table S1 shows a consistent pattern: polymers containing hydrolysable ester bonds are far more likely to reach advanced transformation endpoints than polymers composed predominantly of carbon–carbon backbones. In practice, polymer chemistry largely determines whether enzymatic activity can progress beyond surface modification toward depolymerization, microbial assimilation, and ultimately polymer-derived carbon conversion. Polyolefins such as polyethylene (PE) and polypropylene (PP), together with polymers including polystyrene (PS) and polyvinyl chloride (PVC), possess chemically stable carbon–carbon backbones that confer exceptional resistance to enzymatic attack while limiting substrate accessibility [22,36]. Unlike hydrolysable polyesters, these polymers contain few functional groups that can be specifically recognized by enzymes, restricting most biological activity to surface oxidation or other physicochemical modifications.

Polymer composition is only part of the explanation. Structural characteristics such as crystallinity, molecular weight, and hydrophobicity further influence transformation efficiency. High crystallinity reduces chain mobility and limits enzyme penetration into the polymer matrix, whereas elevated molecular weight decreases the likelihood of complete chain cleavage. Strong hydrophobicity likewise restricts enzyme adsorption and microbial attachment, reducing effective contact between enzymes and polymer surfaces. As a result, enzymatic activity is generally confined to amorphous regions or pre-existing surface defects, while highly ordered crystalline domains remain largely inaccessible [17,21].

These structural characteristics also help explain the considerable variability reported among studies investigating the same polymer. Differences in crystallinity, molecular-weight distribution, additive composition, manufacturing processes, and previous environmental weathering can markedly influence enzyme accessibility and transformation efficiency. Apparently contradictory results obtained for identical polymer types therefore often reflect differences in substrate properties rather than intrinsic differences in enzymatic performance [22,36].

Viewed within the context of the ELF, successful enzyme-mediated transformation depends not only on catalytic capability but also on the intrinsic susceptibility of the polymer substrate. Polymer chemistry establishes the upper boundary of biologically achievable transformation and largely determines whether enzymatic processes can progress beyond the lower evidence levels of the framework. The next subsection examines how environmental weathering modifies these physicochemical barriers and, in doing so, influences subsequent enzyme accessibility.

### 5.3. Environmental Weathering Facilitates but Does Not Demonstrate Polymer Transformation

Environmental weathering is one of the principal processes influencing enzyme-mediated plastic transformation because it modifies the physicochemical properties of polymer surfaces before biological colonization begins. Although weather alone rarely results in complete polymer degradation, it often determines whether enzymes gain access to susceptible regions of the polymer and whether transformation can progress beyond the earliest ELF categories. Differences among many of the studies summarized in Supplementary Table S1 therefore reflect variations in substrate weathering history as much as differences in enzyme performance.

Exposure to ultraviolet radiation, thermal cycling, mechanical abrasion, and oxidative environmental conditions progressively alters polymer surfaces by generating microcracks, increasing roughness, introducing oxygen-containing functional groups, and reducing surface hydrophobicity [22,36]. These modifications increase the surface area available for microbial attachment, facilitate enzyme adsorption, and improve water penetration into the polymer matrix. Weathered plastics consequently become more susceptible to subsequent biological transformation than pristine materials, even when exposed to the same enzyme systems. Such physicochemical alterations should not, however, be interpreted as evidence of biodegradation. Most abiotic weathering processes remain confined to surface oxidation and physicochemical aging without producing sufficient polymer backbone cleavage to support microbial assimilation or polymer-derived carbon conversion [18,21,36]. Their primary role is to reduce the physicochemical barriers that initially limit enzyme accessibility, making abiotic weathering and biological transformation complementary rather than interchangeable processes.

The extent to which weathering enhances enzyme-mediated transformation depends strongly on polymer chemistry. Polyolefins generally require prolonged environmental exposure before measurable increases in enzyme accessibility occur because their chemically stable carbon–carbon backbones remain largely unaffected by mild oxidative processes. By contrast, polymers containing hydrolysable ester bonds often require substantially less preconditioning before becoming susceptible to enzymatic attack. These differences reinforce the central role of polymer chemistry in determining transformation efficiency, while weathering primarily modifies substrate accessibility. Weathering also promotes establishment of the plastisphere by creating surface conditions favorable for microbial attachment and biofilm development. Increased surface roughness, higher surface energy, and the introduction of oxygen-containing functional groups enhance microbial colonization and facilitate localization of extracellular enzymes at the polymer interface [2,38]. Even so, enhanced colonization should not be equated with polymer depolymerization, highlighting the distinction between microbial attachment, enzyme accessibility, and experimentally validated polymer transformation.

Within the context of the ELF, environmental weathering is best viewed as a process that modifies substrate accessibility rather than direct evidence of biodegradation. By reducing physicochemical barriers at the polymer surface, it facilitates progression through the earliest stages of the framework. Advancement toward depolymerization, microbial assimilation, and polymer-derived carbon conversion nevertheless depends on enzyme specificity, microbial metabolism, and rigorous analytical validation. The next subsection examines how these biological interactions develop at the polymer surface and influence subsequent stages of transformation.

### 5.4. Plastisphere, Biofilms, and Enzyme Localization

Formation of the plastisphere represents one of the earliest biological events during enzyme-mediated plastic transformation. Once exposed to natural environments, polymer surfaces are rapidly colonized by diverse microbial communities that attach to the material and produce extracellular polymeric substances (EPS), forming structured biofilms at the polymer–environment interface [2,37,38]. Within the ELF, microbial colonization and biofilm development correspond primarily to the earliest stages of transformation because they demonstrate biological interaction with the polymer without, by themselves, providing evidence of polymer depolymerization or biodegradation.

Although biofilm formation is often regarded as an indicator of plastic degradation, its principal biological function is to establish a stable interface between microorganisms and the polymer surface. This interface promotes prolonged microbial attachment, concentrates extracellular enzymes, retains hydrolytic and oxidative reaction products, and creates localized microenvironments that favor enzymatic activity [21,37,38]. Such conditions markedly increase the likelihood that enzyme-mediated reactions will occur at the polymer surface. Even so, the presence of a mature plastisphere does not demonstrate that polymer chains have undergone depolymerization or that polymer-derived carbon has entered microbial metabolism.

The composition and metabolic potential of plastisphere communities are highly dynamic, varying with polymer chemistry, environmental conditions, nutrient availability, and exposure history. Different polymer types selectively enrich distinct microbial taxa, giving rise to biofilms with different enzymatic capabilities and metabolic profiles [2,37,38]. Successful polymer transformation therefore depends not only on microbial colonization but also on recruitment of communities capable of producing the appropriate extracellular enzymes and metabolizing the transformation products that are generated.

The plastisphere also promotes synergistic interactions among microorganisms. Different microbial populations may contribute complementary metabolic functions, allowing sequential transformation processes in which one organism initiates polymer oxidation or depolymerization while others assimilate and further metabolize the resulting intermediates [21]. Such metabolic cooperation is likely to increase overall transformation efficiency, particularly under environmental conditions where complete polymer degradation is unlikely to be accomplished by a single microorganism or enzyme system.

Despite these advantages, biofilm formation alone rarely enables progression toward higher ELF categories [18,21]. Many of the studies summarized in Supplementary Table S1 describe extensive microbial colonization and well-developed biofilms while providing little or no analytical evidence of polymer depolymerization, microbial assimilation, or polymer-derived carbon conversion. These findings reinforce that plastisphere formation is an important biological prerequisite for enzyme-mediated plastic transformation, but not evidence of biodegradation in the absence of molecular or carbon-resolved validation [18,19]. The plastisphere should therefore be viewed primarily as a biological platform that promotes enzyme localization and facilitates polymer–microorganism interactions rather than as direct evidence of polymer transformation [2,38]. Progression beyond the early ELF categories ultimately depends on whether extracellular enzymatic reactions generate assimilable transformation products and whether microorganisms possess the metabolic capacity to utilize those intermediates. These metabolic constraints are examined in the following subsection.

### 5.5. Microbial Assimilation and Carbon Conversion

The transition from polymer depolymerization to microbial assimilation represents one of the most critical, and least frequently demonstrated steps in enzyme-mediated plastic transformation. Although extracellular enzymes generate soluble oligomers or monomers through polymer chain cleavage, these products contribute to biodegradation only after they are transported into microbial cells, metabolized through intracellular pathways, and ultimately incorporated into biomass or converted to inorganic carbon [20,21,22]. Depolymerization alone therefore cannot be considered evidence of complete biological transformation.

Application of the ELF identifies this transition as one of the principal bottlenecks in the current literature. As shown in Supplementary Table S1, relatively few studies provide direct evidence that polymer-derived transformation products are assimilated by microorganisms, and even fewer demonstrate subsequent conversion of polymer-derived carbon into CO_2_ or biomass [17,18,19,20,21]. This imbalance indicates that the link between extracellular polymer transformation and intracellular metabolism remains insufficiently documented in most enzyme-mediated systems.

Assimilation depends on a series of biological processes extending well beyond extracellular enzyme activity. Polymer-derived intermediates must first cross the microbial membrane through appropriate transport systems before entering central metabolic pathways. Once internalized, they may be incorporated into β-oxidation, aromatic catabolic pathways, the tricarboxylic acid (TCA) cycle, or other metabolic networks, depending on their chemical structure and on the metabolic capabilities of the microorganism involved [20,21,22]. Efficient extracellular depolymerization alone is therefore insufficient if microorganisms lack transport systems or intracellular metabolic pathways required to utilize the released products.

This distinction explains why many studies reporting polymer depolymerization remain confined to intermediate ELF categories. Detection of oligomers or monomers confirms that extracellular enzymatic reactions have cleaved polymer chains but does not establish that these compounds subsequently become biologically available or contribute to microbial growth [21,22]. Likewise, microbial growth observed in the presence of plastics does not necessarily indicate utilization of polymer-derived carbon, since microorganisms may instead metabolize residual additives, plasticizers, low-molecular-weight contaminants, extracellular organic matter, or other carbon sources present within the experimental system [18,21,22].

Direct demonstration of polymer-derived carbon conversion requires analytical approaches capable of tracing carbon flow from the polymer into microbial metabolism. Stable-isotope labeling, isotope-ratio mass spectrometry, respirometry analyses using labeled substrates, and carbon mass-balance approaches currently provide the strongest evidence for microbial assimilation and mineralization because they distinguish polymer-derived carbon from alternative environmental carbon sources [17,18,19,21]. These methodologies therefore underpin the highest ELF categories.

Microbial assimilation provides the essential biological link between extracellular depolymerization and true biodegradation by establishing whether polymer-derived transformation products are incorporated into microbial metabolism rather than merely released into the surrounding environment [20,21,22]. As reflected in Supplementary Table S1, many enzyme systems successfully initiate polymer transformation through surface modification or depolymerization, yet only a small proportion of studies provide direct evidence that these processes advance to microbial assimilation and ultimately to polymer-derived carbon conversion [9,18,19].

This progression represents the central conceptual contribution of the ELF. The framework emphasizes that the strength of transformation claims depends not simply on the occurrence of enzymatic reactions, but on the quality of the analytical evidence supporting each successive stage of polymer transformation. By distinguishing surface modification, depolymerization, microbial assimilation, and polymer-derived carbon conversion as progressively validated biological endpoints, the ELF establishes objective criteria for interpreting transformation claims and provides a common basis for improving the reliability, comparability, and biological relevance of future studies. Future advances in enzyme-mediated plastic transformation will therefore depend as much on rigorous analytical validation as on the discovery and optimization of new plastic-transforming enzymes.

## 6. Challenges and Perspectives

The systematic evaluation performed in this review shows that relatively few studies progressed beyond intermediate evidence levels despite the rapid expansion of research on enzyme-mediated plastic transformation. The major challenge is no longer identifying microorganisms or enzymes capable of interacting with synthetic polymers but demonstrating that these interactions culminate in biologically meaningful polymer transformation. Establishing direct evidence of microbial assimilation and polymer-derived carbon conversion therefore remains the principal objective for the next generation of studies.

Progress toward this goal will require greater methodological consistency across the field. Harmonized experimental protocols, appropriate abiotic controls, quantitative carbon mass-balance approaches, standardized polymer characterization, and clearly defined minimum analytical requirements for each transformation stage would substantially improve reproducibility while facilitating meaningful comparisons among independent studies. Standardization is equally important for reducing the inconsistent interpretation of biodegradation that still characterizes much of the current literature.

A second priority is the broader integration of complementary analytical techniques capable of resolving carbon flow throughout the entire transformation process. Radioactive or stable isotope labeling, isotope-ratio mass spectrometry, metabolomics, compound-specific analyses, and quantitative carbon mass-balance approaches should increasingly be combined with conventional physicochemical characterization to establish direct links between extracellular depolymerization, microbial assimilation, intracellular metabolism, and polymer-derived carbon conversion.

Future research should also move beyond simplified laboratory systems toward environmentally relevant conditions that better reflect the complexity of natural ecosystems. Polymer mixtures, weathered plastics, commercial additives, multispecies microbial communities, and long-term transformation experiments are likely to provide a more realistic understanding of enzyme-mediated plastic transformation. At the same time, advances in enzyme engineering, synthetic biology, systems microbiology, and analytical chemistry offer new opportunities to investigate the interactions among polymer chemistry, microbial metabolism, and environmental conditions with substantially greater mechanistic resolution.

The ELF was developed as a practical tool for interpreting experimental evidence, but its potential extends beyond the scope of this review. The framework can support experimental design, define minimum evidence requirements, facilitate comparisons among independent studies, and provide a common basis for future systematic reviews and meta-analyses. As analytical methodologies continue to evolve, the ELF can be refined to incorporate emerging technologies while preserving its central principle: plastic biodegradation claims should be evaluated according to the strength of the experimental evidence supporting each stage of biological transformation.

## 7. Conclusions

The systematic evaluation of 102 experimental studies demonstrates that the principal challenge in enzyme-mediated plastic transformation is no longer identifying enzymes capable of interacting with synthetic polymers but generating sufficiently robust analytical evidence to validate successive stages of biological transformation. Microbial colonization, surface modification, depolymerization, transformation-product formation, microbial assimilation, and polymer-derived carbon conversion represent distinct biological endpoints that require progressively stronger experimental validation and should not be regarded as equivalent indicators of biodegradation.

To address this challenge, it was proposed the ELF as a standardized approach for classifying enzyme-mediated plastic transformation according to the highest experimentally validated transformation endpoint. Application of the ELF shows that hydrolysable polyesters currently provide the most analytically tractable benchmark systems for demonstrating advanced transformation, whereas conventional plastics remain largely confined to lower evidence levels because of their intrinsic recalcitrance and the limited use of analytical approaches capable of validating microbial assimilation and polymer-derived carbon conversion.

These findings indicate that future progress will depend less on discovering additional plastic-transforming enzymes than on integrating rigorous analytical methodologies capable of establishing unequivocal links between extracellular polymer modification, microbial metabolism, and the fate of polymer-derived carbon. More broadly, the ELF provides a common framework for interpreting transformation claims, guiding experimental design, improving comparability among independent studies, and promoting more reproducible, mechanistically rigorous, and environmentally relevant research on microbial plastic transformation. Ultimately, progress in the field will be determined not only by what enzymes can do, but by what the experimental evidence can convincingly demonstrate.

## Data Availability

No new data were created or analyzed in this study. Data sharing is not applicable to this article.

## Supplementary Materials

The following supporting information can be downloaded at: https://www.mdpi.com/article/10.3390/microorganisms14081787/s1, Table S1: Complete experimental-study inventory classified according to the Evidence-Level Framework (ELF). Refs. [40,41,42,43,44,45,46,47,48,49,50,51,52,53,54,55,56,57,58,59,60,61,62,63,64,65,66,67,68,69,70,71,72,73,74,75,76,77,78,79,80,81,82,83,84,85,86,87,88,89,90,91,92,93,94,95,96,97,98,99,100,101,102,103,104,105,106,107,108,109,110,111,112,113,114,115,116,117,118,119,120,121,122,123,124,125,126,127,128,129] can be found in Supplementary Materials.

## Abbreviations

BHET, bis(2-hydroxyethyl) terephthalate; CALB, Candida antarctica lipase B; DSC, Differential Scanning Calorimetry; ELF, Evidence-Level Framework; FTIR, Fourier Transform Infrared Spectroscopy; GC–MS, Gas Chromatography–Mass Spectrometry; HPLC, High-Performance Liquid Chromatography; LC–MS, Liquid Chromatography–Mass Spectrometry; LDPE, low-density polyethylene; MHET, mono(2-hydroxyethyl) terephthalate; MnP, manganese peroxidase; NMR, Nuclear Magnetic Resonance; PBAT, polybutylene adipate terephthalate; PET, polyethylene terephthalate; PETase, polyethylene terephthalate hydrolase; PE, polyethylene; PHA, polyhydroxyalkanoates; PLA, polylactic acid; PP, polypropylene; PS, polystyrene; SEC/GPC, Size Exclusion Chromatography/Gel Permeation Chromatography; SEM, Scanning Electron Microscopy; TGA, Thermogravimetric Analysis; TPA, terephthalic acid.
