# Supplementary material for: An Evidence-Level Framework for Evaluating Enzyme-Mediated Plastic and Microplastic Transformation
Source: Microorganisms. 2026 Aug 14;14(8):1787. doi: 10.3390/microorganisms14081787 (PMC13515129; doi:10.3390/microorganisms14081787)
Supplement: Supplementary file 1 [file microorganisms-14-01787-s001.zip › microorganisms-4452632-supplementary.pdf]

Table S1. Complete experimental-study inventory classified according to the Evidence-Level Framework (ELF)

ELF level	Source group	Polymer	Enzyme/ microbial system	Enzyme class	Highest validated evidence	Principal analytical evidence	Key validated conclusion	Main analytical gap	Reference
**ELF LEVEL 0**									
0	Microbial consortium	Marine plastic debris	Plastisphere microbiota	Mixed/unspecified enzymatic system	Microbial colonization	SEM; community sequencing	Microbial colonization of plastic debris was demonstrated.	No direct evidence of polymer modification.	[2]
0	Microbial consortium	PET bottles/ marine plastic debris	Marine biofilms	Mixed/unspecified enzymatic system	Biofilm formation	Community sequencing	Substrate-, season-, and geography-dependent biofilm formation was shown.	No surface alteration or polymer transformation was validated.	[38]
0	Microbial consortium	PE/PS/PP	Plastisphere microorganisms	Mixed/unspecified enzymatic system	Plastic-degradation potential inferred	Metagenomic and phylogenetic screening	Plastic-degrading potential was inferred from taxonomic and functional patterns.	No experimental polymer-transformation endpoint was demonstrated.	[40]
0	Microbial consortium	Marine plastic debris	Deep-sea plastisphere bacterial and archaeal communities	Mixed/unspecified enzymatic system	Long-term microbial colonization	Community profiling; microscopy	Long-term colonization of deep-sea plastic-associated communities was demonstrated.	No direct polymer modification or transformation endpoint was validated.	[41]
0	Microbial consortium	Marine plastic debris	Early marine biofilm community	Mixed/unspecified enzymatic system	Early biofilm formation	Microscopy; microbial abundance	Rapid microbial biofilm formation on marine plastic debris was demonstrated.	No direct evidence of polymer alteration or degradation.	[42]
**ELF LEVEL 1**									
1	Enzyme system	PE microplastics	*Zalerion maritimum*	Not specified	Surface physico-chemical modification	SEM; FTIR; weight loss	Fungal exposure induced surface and physicochemical changes in PE microplastics.	No molecular-weight or product-based evidence of chain scission.	[43]
1	Microbial consortium	PE microplastics	Mixed landfill microbial consortium	Mixed/unspecified enzymatic system	Indirect polymer alteration	SEM; weight loss	Microbial colonization and apparent PE particle alteration were reported.	No molecular-weight analysis or transformation-product identification.	[44]
1	Bacteria	PE	Cold-adapted bacterial laccase from *Psychrobacter* sp. NJ228	Laccase	Surface oxidation	SEM; FTIR	A cold-adapted laccase system produced detectable PE surface oxidation.	No molecular validation of depolymerization.	[45]
1	Enzyme system	PE microplastics	Cell-surface-displayed cold-active laccase	Laccase	Improved enzyme-polymer interaction and surface modification	SEM; FTIR; polymer characterization	Surface-displayed laccase enhanced low-temperature PE microplastic modification.	No direct chain-cleavage or carbon-fate evidence.	[46]
1	Fungi	HDPE	*Bjerkandera adusta* TBB-03 cultivated with lignocellulosic substrates	Ligninolytic oxidative system (laccase-associated)	Advanced surface and structural alteration	SEM; Raman spectroscopy; laccase activity	Co-cultivation with lignocellulosic substrates stimulated fungal growth and ligninolytic activity, promoting cracks, oxidation, and structural modifications on HDPE surfaces.	No molecular-weight reduction, polymer-derived degradation products, microbial assimilation, or isotope-based carbon mineralization were demonstrated.	[47]
1	Fungi	Synthetic plastics	Extracellular lipase from *Aspergillus niger*	Lipase	Surface modification	SEM; FTIR; weight loss	Extracellular lipase treatment was associated with surface and chemical changes in plastics.	No carbon-tracking or product-based evidence.	[48]

1	Fungi	LDPE	Filamentous fungi from landfill plastic waste	Not specified	Surface oxidation and morphological alteration	Respiration tests; SEM; FTIR	Several fungal strains caused strain-dependent oxidation and surface damage in LDPE films.	No quantitative chain-scission, product, or carbon-tracking evidence.	[49]
1	Fungal-bacterium consortium	LDPE	*Bacillus* EBL50 + *Sarocladium* EBL 60	Not specified	Surface alteration	SEM, FTIR	Co-cultures were more efficient than isolated microorganisms	No molecular-weight or carbon-flow evidence.	[50]
1	Bacteria	PE	Engineered *Bacillus velezensis* delivering PE-group-degrading enzyme	Not specified	Advanced polymer alteration	Functional-group analysis; weight loss	Engineered bacterial delivery increased PE functional-group modification and apparent degradation.	Quantitative chain scission and carbon fate remained unresolved.	[51]
1	Microbial consortium	PET e PS	Native mangrove microbial consortium	Mixed/unspecified enzymatic system	Colonization-enhanced surface alteration	Weight loss, surface alterations	Up to 1.6% weight loss	No direct evidence of polymer-chain cleavage or product formation.	[52]
1	Fungi	PE, PCL, PS and PLA	*Clonostachys rosea*	Cutinases	Colonization-enhanced surface alteration	SEM, genetic prospection	Halo formation and identification of cutinase genes	No direct evidence of polymer-chain cleavage or product formation.	[53]
1	Fungi	PEBD	*Pleurotus ostreatus; Agaricus bisporus*	Laccases and MnP	Colonization-enhanced surface alteration	SEM, FTIR, qPCR	Colonization and superficial alterations	No direct evidence of polymer-chain cleavage or product formation.	[54]
1	Bacteria	PEAD	*Bacillus cereus, Bacillus subtilis*	Oxidoreductases	Colonization-enhanced surface alteration	FTIR, SEM, SDS-PAGE	Up to 3.8% weight loss in 30 days	No direct evidence of polymer-chain cleavage or product formation.	[55]
1	Bacteria	PEAD / PEBD	Enriched community (Proteobacteria, Actinobacteriota, and Bacteroidota)	Mixed/unspecified enzymatic system	Colonization-enhanced surface alteration	ATR-FTIR, SEM, weight loss	Up to 5.6% weight loss	No direct evidence of polymer-chain cleavage or product formation.	[56]
1	Bacteria	LDPE microplastics	Biofilm-forming bacterial system	Mixed/unspecified enzymatic system	Colonization-enhanced surface alteration	Biofilm assays; SEM; FTIR	Enhanced bacterial colonization and biofilm formation were associated with LDPE surface modification.	No direct molecular-weight decrease or polymer-derived product identification.	[57]
1	Bacteria	PE and PP	Four mangrove-derived bacterial strains	Mixed/unspecified enzymatic system	Biodegradation potential and surface modification	Growth screening; biofilm; FTIR/SEM	Mangrove isolates showed polymer colonization and preliminary PE/PP alteration potential.	Chain scission and polymer-derived products were not directly validated.	[58]
1	Bacteria	Marine microplastics	Biofilm-forming marine bacterial isolates	Mixed/unspecified enzymatic system	Microplasti-sphere colonization and surface alteration	Biofilm assays; microscopy; FTIR	Marine isolates formed biofilms and produced detectable surface-level changes in microplastics.	No molecular-weight, product, assimilation, or carbon-fate evidence.	[59]
1	Bacteria	PET	Sediment-associated bacterial isolates	Mixed/unspecified enzymatic system	Potential growth on PET	Growth screening; community identification	Bacterial isolates displayed growth potential in the presence of PET.	Direct PET chain cleavage and product formation were not established.	[60]
1	Enzyme system	PET	Protease-based PET interaction system	Neprilysin; cutinase-like enzyme	Enzyme–polymer interaction and predicted cleavage potential	Structural modelling; molecular simulation	Two proteases displayed mechanistic potential for PET-chain interaction and hydrolysis.	Experimental polymer depolymerization and product recovery were not conclusively validated.	[61]

1	Enzyme system	PET	Surface-active hydrolytic enzymes	Hydrolases	Enzymatic surface modification	Surface chemistry; wettability; microscopy	Enzyme treatment modified PET surface properties.	No validated bulk depolymerization, monomer recovery, or assimilation.	[62]
**ELF LEVEL 2**									
2	Fungi	PE-MPs	*Aspergillus niger*	Laccases, cutinases	Biofragmentation	FTIR, SEM, GC-MS, XRD	Up to 7.65 ± 0.92% of weight loss in 30 days	No unequivocal chain-scission, assimilation, or carbon-fate evidence.	[63]
2	Fungi	PE, PET e PS	*Aspergillus niger, Candida albicans, Acremonium sclerotigenum*	Lacases, esterases	Biofragmentation	Biofilm formation, FTIR, SEM, weight loss	Up to 16% (PE)	No unequivocal chain-scission, assimilation, or carbon-fate evidence.	[64]
2	Fungi	PEBD	*Aspergillus* spp., *Penicillium* spp., *Trichoderma* spp.	Laccases, esterases and peroxidases	Biofragmentation	SEM, FTIR, weight loss	Up to 47% weight loss	No unequivocal chain-scission, assimilation, or carbon-fate evidence.	[65]
2	Fungi	PVC, PS e PMMA	*Lecanicillium coprophilum*	Oxidoreductases	Biofragmentation	Fluorescence, SEM, biomass	Up to 17,3% OS degradation	No unequivocal chain-scission, assimilation, or carbon-fate evidence.	[66]
2	Fungi	PE e PP	*Aspergillus niger, Cunninghamella elegans* and *Aspergillus flavus*	Laccases, peroxidases	Biofragmentation	FTIR, SEM, GC-MS, XRD	Up to 56% (PE) and 44% (PP)	No unequivocal chain-scission, assimilation, or carbon-fate evidence.	[67]
2	Microbial consortium	PVC	*Priestia megaterium* (SXC01 and 1MB38) + *Bacillus cereus* (1MM03)	Esterases, oxidoredutases	Biofragmentation	Enzyme production, biofilm formation, weight loss	Higher enzymatic production and adhesion to PVC compared to individual isolates.	No unequivocal chain-scission, assimilation, or carbon-fate evidence.	[68]
2	Bacteria	LDPE microplastic	*Bacillus subtilis* V8, *Pseudomonas aminophilus* B1-4, among other	Mixed/unspecified enzymatic system	Biofragmentation	CO_2 evolution; weight loss; SEM; FTIR; electrical conductivity	Bacterial strains promoted LDPE biofragmentation, evidenced by CO_2 evolution, weight loss, and physicochemical surface alterations.	No molecular-weight analysis, degradation-product identification, microbial assimilation, or isotope-resolved carbon tracing.	[69]
2	Bacteria	PE	*Pseudomonas aeruginosa* and *Rossellomorea aquimaris*	Oxidoreductases	Biofragmentation	FTIR, GC-MS, weight loss	Up to 22.5% of weight loss after 60 days	No unequivocal chain-scission, assimilation, or carbon-fate evidence.	[70]
2	Fungi	Microplastics of printed circuit boards	*Penicillium brevicompactum*	Oxidoreductases	Biofragmentation	ATR-FTIR, microscopy, weight loss	Up to 75% of weight loss after 28 days	No unequivocal chain-scission, assimilation, or carbon-fate evidence.	[71]
2	Bacteria	PET	*Comamonas testosteroni* F6	Esterases	Initial biofragmentation	HPLC, FTIR, DSC, SEM	Up to 11.0% of weight loss in 5 days	No unequivocal chain-scission, assimilation, or carbon-fate evidence.	[72]
2	Microbial consortium	PP	*Serratia marcescens* + *Bacillus tropicus; Serratia marcescens* + *Bacillus cereus; Serratia marcescens* +	Extracellular hydrolases (protease, lipase, esterase) and oxidoreductase-related activity	Biofragmentation	Weight loss; SEM; FTIR; 1H NMR; metabolite profiling	Three consortium configurations were tested: 63.0 ± 2.8%, 72.8 ± 1.1%, and 79.5 ± 2.1% mass loss in 28 days; the three-member consortium showed the highest apparent efficiency.	No direct molecular-weight, product, assimilation, or carbon-fate validation.	[73]

			Bacillus tropicus + *Bacillus cereus*						
2	Bacteria	PET	*Bacillus paramycoides, B. cereus, B. safensis, B. luciferensis*	Esterases Lipases	Initial biofragmentation	SEM, FTIR, weight loss	2% weight loss in 20 days	No unequivocal chain-scission, assimilation, or carbon-fate evidence.	[74]
2	Microbial consortium	PEBD, PET and PEAD	*Bacillus licheniformis* + *Bacillus altitudinis*	Hydrolases e oxidoredutases	Biofragmentation	FTIR, weight mass, toxicity	PEBD (19.4%), PET (6.0%) and PEAD (2.6%), with greater efficiency after pre-treatment	No unequivocal chain-scission, assimilation, or carbon-fate evidence.	[75]
2	Bacteria	PE, PVC and PET	*Pseudomonas aeruginosa, Bacillus flexus, Pseudomonas azotoformans*	Oxidoreductases + Hydrolases	Biofragmentation	Weight loss	Up to 37.9% depending on the polymer	No unequivocal chain-scission, assimilation, or carbon-fate evidence.	[76]
2	Bacteria	PE	*Lysinibacillus macroides*	Oxidoreductases	Biofragmentation	SEM, FTIR, mass loss	40% in 40 days	No unequivocal chain-scission, assimilation, or carbon-fate evidence.	[77]
2	Fungi	PS	*Pleurotus eryngii; Trametes versicolor*	Laccases, MnP, LiP	Biofragmentation	FTIR, phenolics,enzym-atic activity	Evidence on superficial oxidation	No unequivocal chain-scission, assimilation, or carbon-fate evidence.	[78]
2	Microbial consortium	PE-MPs e nylon-MPs	Bacterial consortium dominated by *Pulmonis* sp.	Mixed/unspecified enzymatic system	Biofragmentation	Weight loss	Performance similar to that of the isolate for PE-MPs and nylon-MPs, indicating an interaction dependent on the composition of the consortium.	No unequivocal chain-scission, assimilation, or carbon-fate evidence.	[79]
2	Bacteria	PP	*Bacillus pasteurii*	Oxidoreductases	Biofragmentation	SEM, weight loss	23.2% of weight loss	No unequivocal chain-scission, assimilation, or carbon-fate evidence.	[80]
2	Bacteria	PP	*Streptomyces ardesiacus* NBIO111	Oxidoreductases	Biofragmentation	FTIR, GC-MS	17.5% of weight loss in 90 days	No unequivocal chain-scission, assimilation, or carbon-fate evidence.	[81]
2	Bacteria	PVC microplastics	*Streptomyces gobitricini*	Hydrolases/oxidoreductases (putative)	PVC biofragmentation	Mass loss; FTIR; SEM	The actinobacterial strain induced physicochemical and morphological changes in PVC microplastics.	No unequivocal molecular-weight decrease, assimilation, or carbon-fate evidence.	[82]
2	Fungi	PS and PVC microplastics	*Candida parapsilosis* after AOP pretreatment	Mixed/unspecified enzymatic system	Pretreatment-assisted biofragmentation	AOP treatment; weight loss; FTIR; microscopy	AOP pretreatment increased the susceptibility of PS and PVC microplastics to fungal alteration.	Polymer-derived products and carbon conversion were not directly resolved.	[83]
2	Fungi	PET	*Trametes versicolor* and *Pleurotus sajor-caju*	Laccases; peroxidases; hydrolases (putative)	Fungal biofragmentation	Weight loss; FTIR; SEM; enzyme activity	Both white-rot fungi produced strain-dependent PET surface and chemical alterations.	Direct monomer identification, assimilation, and carbon balance were not established.	[84]
2	Fungi	LDPE	*Pleurotus ostreatus, Pleurotus eryngii, Trametes hirsuta, Trametes versicolor*	Laccases, MnP, LiP	biofragmentation	Weight loss; FTIR; SEM; contact angle; tensile strength; ligninolytic enzyme assays	Several white-rot fungi induced physicochemical modifications of LDPE, including surface oxidation, changes in hydrophobicity and mechanical properties, accompanied by ligninolytic enzyme production.	Multiple complementary analyses demonstrated surface deterioration and biofragmentation of LDPE. However, no direct evidence of molecular-weight reduction (e.g., GPC/SEC), soluble	[85]

							P. ostreatus and *T. versicolor* showed the highest degradation potential.	depolymerization products, carbon assimilation, or mineralization was provided.	
2	Bacteria	LDPE	*Alcaligenes faecalis* ISJ128	Oxidoreductases (putative)	LDPE biofragmentation	Weight loss; FTIR; SEM	The indigenous strain caused measurable mass loss and oxidative surface changes in LDPE.	No direct molecular-weight distribution or polymer-carbon tracking.	[86]
2	Bacteria	PET	*Rhizobacterial* isolates	Hydrolases (putative)	PET biodeterioration/ biofragmentation	Weight loss; FTIR; SEM	Rhizobacteria caused detectable physicochemical alteration of PET.	Monomer formation and microbial assimilation were not directly validated.	[87]
2	Bacteria	LDPE films	*Bacillus cereus* NJD1	Oxidoreductases/ hydrolases (putative)	LDPE biofragmentation without pretreatment	Weight loss; FTIR; SEM	*B. cereus* NJD1 altered untreated LDPE films and produced measurable apparent degradation.	No unequivocal chain-scission products or carbon-fate evidence.	[88]
2	Bacteria	PS and PP	Deep-sea *Pseudoaltero-monas* spp.	Mixed/unspecified enzymatic system	Polymer fragmentation	Microscopy; spectroscopy; particle-size analysis	Psychrophilic bacteria altered PS and PP while generating smaller plastic particles.	Fragmentation was shown, but assimilation and mineralization were not resolved.	[89]
2	Bacteria	PET	Environmental microbial isolates	Hydrolases (putative)	In vivo PET biodeterioration	Weight loss; FTIR; SEM	Environmental isolates produced measurable PET alteration under *in vivo* exposure conditions.	Polymer-derived monomers and carbon conversion were not directly demonstrated.	[90]
2	Bacteria	LDPE	*Paenibacillus* sp. and *Serratia* sp.	Oxidoreductases/ hydrolases (putative)	LDPE biofragmentation	Weight loss; FTIR; microscopy	Marine-soil isolates caused physicochemical and surface changes in LDPE.	No direct product identification, assimilation, or carbon tracing.	[91]
2	Bacteria	Polyester microfibers	*Exiguobacterium* sp.	Hydrolases (putative)	Polyester microfiber biofragmentation	Weight loss; FTIR; microscopy	The bacterial isolate altered polyester microfibers and produced measurable apparent degradation.	No definitive monomer recovery or polymer-carbon fate analysis.	[92]
2	Bacteria	HDPE microplastic	Selected bacterial isolates under optimized process conditions	Mixed/unspecified enzymatic system	Process-dependent HDPE/ biofragmentation	Weight loss; FTIR; SEM; process optimization	Bacterial performance and cultivation parameters influenced HDPE microplastic alteration.	Direct chain-scission products, assimilation, and carbon balance were not resolved.	[93]
2	Microbial consortium	PP and PS microplastic	Novel microbial consortia	Mixed/unspecified enzymatic system	Consortium-mediated biofragmentation	Weight loss; FTIR; microscopy	Microbial consortia produced measurable physicochemical alteration of PP and PS microplastics.	No unequivocal product, assimilation, or carbon-fate evidence.	[94]
2	Fungi	LDPE	*Cladosporium cladosporioides* Clc/1	Oxidoreductases (putative)	biodeterioration/ biofragmentation	Weight loss; FTIR; SEM	The fungal strain produced oxidative and morphological changes in LDPE.	Molecular-weight decrease and polymer-derived products were not directly resolved.	[95]
2	Bacteria	LDPE and PVC microplastic	*Achromobacter denitrificans* Ebl13 after thermo-oxidative pretreatment	Mixed/unspecified enzymatic system	Pretreatment-assisted biofragmentation	Weight loss; FTIR; SEM; product screening	Thermo-oxidative pretreatment enhanced bacterial alteration of LDPE and PVC microplastics.	Assimilation and polymer-carbon mineralization were not directly validated.	[96]
2	Microbial consortium	Wastewater microplastic	Activated-sludge/effluent microbial community	Mixed/unspecified enzymatic system	Microplastic abundance and physicochemical alteration	Particle quantification; microscopy; spectroscopy	Wastewater exposure was associated with changes in microplastic abundance and characteristics.	Direct chain-scission, product, and carbon-fate evidence remained unresolved.	[97]

2	Bacteria	PE	*Gordonia polyisopreni-vorans* B251	Mixed/unspecified enzymatic system	Biofilm-enhanced biofragmentation	Biofilm assays; weight loss; FTIR; SEM	Biofilm formation increased PE surface oxidation and apparent biodegradation.	No direct polymer-derived carbon assimilation or mineralization.	[98]
2	Bacteria	Plastic films	*Alcanivorax* enrichment cultures	Mixed/unspecified enzymatic system	Temperature-dependent surface alteration and fragmentation	Enrichment; microscopy; spectroscopy; product screening	*Alcanivorax* altered plastic-film morphology and released low-molecular-weight compounds under marine conditions.	Complete product attribution, assimilation, and carbon balance were not established.	[99]
**ELF LEVEL 3**									
3	Enzyme system	PET and PEF	Wild-type and engineered *Ideonella sakaiensis* PETase	PETase	Enzymatic polyester depolymerization with soluble-product formation	Product-release assays; HPLC-based product analysis; structural analysis; crystallography; SEM	Wild-type and engineered PETase variants hydrolyzed PET and PEF, and active-site engineering improved the degradation of aromatic polyesters.	No microbial assimilation, isotope-resolved carbon tracing, or mineralization of polymer-derived carbon.	[29]
3	Enzyme system	PET	PETase combined with fungal hydrophobin RolA	PETase	Enhanced enzymatic depolimerization	Weight loss; PET hydrolysis assays; HPLC product analysis; surface-wetting analysis	The fungal hydrophobin RolA enhanced PETase adsorption onto PET surfaces, significantly increasing PET hydrolysis and the release of soluble degradation products through improved enzyme–substrate interaction.	No microbial assimilation, isotope-resolved carbon tracing, or complete mineralization of polymer-derived carbon was demonstrated.	[100]
3	Enzyme system	PET	Solid-degrading cutinase + reaction intermediate-hydrolyzing carboxylesterase	Cutinase; Carboxylesterase	Synergistic enzymatic depolymerization with high-purity TPA production	HPLC; TPA quantification; hydrolysis assays; enzyme synergy analysis	The synergistic action of a PET-active cutinase and a carboxylesterase efficiently converted PET into high-purity terephthalic acid by preventing accumulation of hydrolysis intermediates and enhancing overall depolymerization.	No microbial assimilation, isotope-resolved carbon tracing, or complete mineralization of polymer-derived carbon was demonstrated.	[101]
3	Enzyme system	PBAT (poly(butylene adipate-co-terephthalate)	*Fusarium solani* cutinase	Cutinase	Enzymatic hydrolysis with product formation	SEM; ATR-FTIR; XRD; TGA; LC-MS/MS; weight loss; hydrolysis assays	*Fusarium solani* cutinase efficiently hydrolyzed PBAT films, producing soluble oligomers and monomers with concentration-dependent polymer degradation and marked physicochemical changes.	No microbial assimilation, isotope-resolved carbon tracing, or complete mineralization of polymer-derived carbon was demonstrated.	[102]
3	Enzyme system	PET microplastics	PETase in human serum	PETase	Depolymerization under biological matrix conditions	HPLC; NMR spectroscopy; hydrolysis assays	PETase efficiently depolymerized PET microplastic particles into terephthalic acid and MHET in human serum, demonstrating that enzymatic PET hydrolysis remains effective in a complex biological matrix.	No microbial assimilation, isotope-based carbon tracing, or complete mineralization of polymer-derived carbon was demonstrated.	[103]
3	Enzyme system	PET/PEF	Engineered PETase	PETase/MHETase	Polyester depolymerization	Structural analysis; hydrolysis assays	Engineering improved aromatic polyester hydrolysis.	No metabolic validation of hydrolysis products.	[31]
3	Enzyme system	PET	PETase/MHETase two-enzyme system	PETase/MHETase	Synergistic depolymerization	HPLC; LC-MS; structural analysis	Two-enzyme PETase/MHETase action enhanced PET depolymerization.	No carbon-tracing evidence.	[30]

3	Enzyme system	PET	Engineered leaf-branch compost cutinase	Cutinase	High-efficiency monomer recovery	Monomer recovery; process-scale hydrolysis	An engineered PET depolymerase enabled high PET conversion into monomers.	No biological assimilation step.	[35]
3	Fungi	PET	Candida antarctica lipase B + *Humicola insolens* cutinase	Cutinase; Lipase	Enzymatic depolymerization with monomer release	HPLC; terephthalic acid (TPA) quantification; enzyme hydrolysis assays	Synergistic action of CALB and cutinase significantly enhanced PET hydrolysis and terephthalic acid release.	No microbial assimilation, isotope tracing, or polymer-derived carbon mineralization.	[34]
3	Fungi	Polyethylene	*Alternaria alternata* FB1	Mixed/unspecified enzymatic system	Advanced oxidation and depolymerization evidence	SEM; FTIR; XRD; GPC (molecular-weight analysis); GC-MS; transcriptomic analysis	Polyethylene films showed oxidative surface modification, molecular-weight reduction, and degradation products, confirming polymer depolymerization. Transcriptomics identified putative degradation-related genes.	Microbial assimilation of polymer-derived carbon and isotope-resolved mineralization were not demonstrated.	[104]
3	Fungi	PE microplastic particle	*Aspergillus flavus* PEDX3 from *Galleria mellonella* gut	Putative laccase-like multicopper oxidases (LMCOs)	Surface oxidation and partial depolymerization	FTIR; HT-GPC; RT-PCR	PE microplastic particles showed carbonyl and ether group formation, significant reductions in molecular weight (Mw and Mn), and upregulation of two LMCO genes after fungal incubation.	Direct evidence of polymer depolymerization through molecular-weight reduction and oxidative modification. However, polymer-derived degradation products, microbial carbon assimilation, and mineralization were not directly demonstrated.	[105]
3	Enzyme system	PET	Leaf-branch compost cutinase homolog	Cutinase	PET hydrolysis products	Hydrolysis assays; product analysis	A cutinase homolog released PET hydrolysis products.	No biological assimilation or carbon balance.	[106]
3	Enzyme system	Nano-PET	Magnetic silica-coated immobilized cutinase	Cutinase	Nano-PET hydrolysis	Product analysis; immobilized-enzyme performance	Immobilized cutinase efficiently hydrolyzed nano-PET.	No microbial assimilation or carbon-flow evidence	[107]
3	Bacteria	PET	*Stenotrophomonas pavanii* expressing PETase	PETase	Depolymerization	HPLC, FTIR, biofilm	PET degradation products	No direct validation of microbial assimilation or polymer-carbon conversion.	[108]
3	Bacteria	PET	*Escherichia coli* BL21(DE3) expressing PETase	PETase	Depolymerization	HPLC, FTIR, SEM	Total PET hydrolysis in 36 h	No direct validation of microbial assimilation or polymer-carbon conversion.	[109]
3	Bacteria	PET-MPs	*Stenotropho-monas pavanii* genetically engineered	FastPETase	Depolymerization with product formation	HPLC, FTIR, SEM	Degradation of 63.3%	No direct validation of microbial assimilation or polymer-carbon conversion.	[110]
3	Bacteria	PS	*Gordonia* sp. PS3	Oxidoreductases	Biofragmentation	FTIR, TGA, GPC	Degradation of 33.6% in 40 days	No direct validation of microbial assimilation or polymer-carbon conversion.	[111]
3	Bacteria	PET-MPs	*Priestia megaterium* Jou-S14	Hydrolases	Formation of degradation products	FTIR, LC-MS, XRD	Degradation of 12.1% in 90 days	No direct validation of microbial assimilation or polymer-carbon conversion.	[112]
3	Fungi	PET microplastic	*Candida tropicalis*	Hydrolases (putative)	PET depolymerization	Weight loss; FTIR; SEM;	PET microplastic treatment generated degradation-associated	Direct polymer-carbon assimilation and	[113]

					with metabolite formation	metabolite analysis; toxicity testing	metabolites with subsequent biocompatibility evaluation.	mineralization were not demonstrated.	
3	Enzyme system	PE microplastic	Recombinant *Pichia pastoris* and purified laccase	Laccase	Partial oxidative depolymerization	FTIR; GC-MS; molecular-weight analysis	Synergistic treatment promoted oxidative depolymerization of PE microplastics, evidenced by molecular-weight reduction and degradation products.	No microbial assimilation, isotope-resolved carbon tracing, or complete mineralization of polymer-derived carbon was demonstrated.	[114]
3	Bacteria	PET microplastics	*Stenotropho-monas pavanii* with surface-targeted PETase	PETase	Targeted enzymatic depolymerization	HPLC; FTIR; SEM; enzyme localization	Surface aggregation of PETase improved PET microplastic hydrolysis and product formation.	Microbial assimilation and carbon conversion were not directly validated.	[115]
3	Fungus	PET microplastic	Yeast-based degradation system with Tween 20	Mixed/unspecified enzymatic system	Depolymerization with by-product formation and metabolic pathway prediction	Weight loss; FTIR; SEM; by-product analysis; metabolic pathway prediction	Tween 20 significantly enhanced yeast-mediated degradation of PET microplastics, leading to detectable degradation by-products and prediction of downstream metabolic pathways associated with PET transformation.	Direct experimental validation of microbial assimilation, isotope-resolved carbon flow, and complete mineralization of PET-derived carbon was not demonstrated.	[116]
3	Microbial consortium	PET microplastic	Sequential natural microbial consortia	PETases/hydrolases (putative)	Depolymerization and detoxification	Product analysis; spectroscopy; ecotoxicity assays	Sequential consortia generated PET-related transformation products and reduced associated toxicity.	Carbon-resolved assimilation and mineralization were not established.	[117]
3	Bacteria	PET microplastic	Whole-cell biocatalyst with interface activation	PETase-like hydrolase	Enhanced PET depolymerization	Hydrolysis assays; product analysis; surface characterization	Interface activation improved whole-cell PET microplastic hydrolysis and release of degradation products.	Polymer-derived carbon assimilation was not directly validated.	[118]
3	Bacteria	PET	Engineered *Vibrio natriegens* living biocatalyst	PETase/MHETase system	In situ PET depolymerization	Product quantification; whole-cell biocatalysis assays	Engineered *V. natriegens* enabled in situ PET hydrolysis in seawater-like conditions.	Complete carbon balance and mineralization were not resolved.	[119]
3	Bacteria	PET	Wastewater bacteria engineered by conjugation	PETase-like hydrolase system	Engineered whole-cell PET depolymerization	Hydrolysis assays; product analysis; genetic validation	Conjugation-generated wastewater bacteria acquired PET-depolymerizing capacity.	Assimilation and isotope-resolved polymer-carbon conversion were not demonstrated.	[120]
3	Bacteria	PET	*Bacillus safensis* YX8	Hydrolases (putative)	PET depolymerization and product formation	FTIR; SEM; HPLC/GC-MS; genomic analysis	*B. safensis* YX8 altered PET and generated identifiable degradation-associated products.	Complete assimilation and carbon-resolved mineralization were not established.	[121]
**ELF LEVEL 4**									
4	Enzyme system	PE	Recombinant small laccase	Small laccase	Depolymerization and biological conversion	GC–MS; GPC; FTIR; NMR; HPLC; LC–MS; product quantification	Recombinant small laccase catalyzed oxidative depolymerization of polyethylene, generating low-molecular-weight oxidation products that were subsequently converted into	Direct evidence of polymer depolymerization (GPC and product identification) followed by biological conversion of depolymerization products	[122]

							polyhydroxyalkanoates (PHA) through microbial biotrans-formation, demonstrating the potential for polyethylene bio-upcycling.	into value-added biopolymers. However, no isotope-based carbon tracing or complete carbon mass balance was performed.	
4	Bacteria	PET	*Ideonella sakaiensis*	Mixed/unspecified enzymatic system	Assimilation of PET-derived intermediates	Product formation; growth/assimila-tion evidence	A bacterium degraded PET and assimilated PET-derived monomers through PETase/MHETase-associated pathways.	Complete carbon-resolved mineralization was not established.	[20]
4	Bacteria	PET-derived TPA and EG	Engineered *Pseudomonas putida* KT2440	Mixed/unspecified enzymatic system	Biological utilization of PET-derived monomers	Metabolic conversion; bioconversion assays	PET-derived monomers were metabolically converted into value-added products.	Isotope-based carbon balance was not provided.	[123]
4	Microbial consortium	PP microplastics	Enriched landfill microbial consortium	PETases/hydrolases and oxidoreductases (metagenome-supported)	Biofragmentation + assimilation + bioconversion (PHB production)	Metagenomic analysis; FTIR; weight loss; PHB quantification; microbial community analysis	The enriched microbial consortium degraded polypropylene microplastics and simultaneously converted polymer-derived carbon into intracellular polyhydroxybutyrate (PHB), supporting biological assimilation and value-added bioconversion.	Complete isotope-based carbon balance and direct mineralization (CO_2 evolution) were not demonstrated.	[124]
4	Microbial consortium	PET	Bacterial consortium from aquatic environments	PETases, esterases	Biofragmentation + assimilation	Biofilm formation, identification of degradation products	Consortium with high potential for PET biodegradation	Polymer-derived carbon mineralization was not resolved.	[125]
4	Microbial consortium	PET-MPs	Enriched landfill consortium	PETases, hidrolases	Biofragmentation + assimilation	Genomics, experimental optimization, FTIR, weight loss	Evidence of degradation via complementary mechanisms among consortium members	Polymer-derived carbon mineralization was not resolved.	[126]
4	Microbial consortium	PS-MPs	Microbial consortium from the Jacarta Bay	Mixed/unspecified enzymatic system	Assimilation	Growth in mineral medium con-taining PS as the sole carbon source	Microbial growth associated to PS consumption	Polymer-derived carbon mineralization was not resolved.	[127]
4	Bacteria	LDPE	LDPE-degrading microorganism with PHA synthesis	Mixed/unspecified enzymatic system	Polymer-derived substrate utilization and bioconversion	Growth; polymer alteration; PHA characterization	LDPE degradation was coupled to microbial synthesis of polyhydroxyalkanoate, supporting biological carbon utilization.	Complete isotope-based carbon balance and mineralization were not provided.	[128]
**ELF LEVEL 5**									
5	Fungi	Polyethylene	*Rhodotorula mucilaginosa*	Not specified	Carbon incorporation into biomass and mineralization	NanoSIMS; isotope tracing	Polyethylene-derived carbon assimilation by a marine yeast was demonstrated.	No major analytical gap for ELF 5.	[10]
5	Fungi	[^{14}C]-polystyrene after ozonation	Ligninolytic fungi	Ligninolytic oxidative system	Carbon-resolved mineralization	[^{14}C]CO_2 evolution	Ozonated radiolabeled polystyrene was mineralized by fungal systems.	No major analytical gap for ELF 5.	[11]

5	Microbial consortium	Biodegradable polyesters	Soil microbial communities	Mixed/unspecified enzymatic system	Carbon-resolved mineralization and biomass incorporation	^{13}C tracing; CO_2 evolution; microbial biomass analysis	Polymer-derived carbon was tracked into CO_2 and microbial biomass.	No major analytical gap for ELF 5.	[19]
5	Bacteria	[^{13}C]-polyethylene	*Rhodococcus ruber*	Mixed/unspecified enzymatic system	Carbon assimilation and mineralization	[^{13}C]CO_2; labelled fatty acids	Stable-isotope assays demonstrated PE-derived carbon conversion into CO_2 and biomass lipids.	No major analytical gap for ELF 5.	[9]
5	Fungi	PS	*Capnodium coffeae*	Ligninolytic oxidative system	Mineralization	Mineralization with ^{13}C, GC-IRMS, PLFA-SIP, FTIR, RMN	Carbon conversion to OS	Highest evidence level; remaining limitations relate mainly to system scope and mass-balance completeness.	[129]

REFERENCES

[2]Zettler, E.R.; Mincer, T.J.; Amaral-Zettler, L.A. Life in the Plastisphere: Microbial Communities on Plastic Marine Debris. *Environ. Sci. Technol.* **2013**, *47*, 7137–7146. https://doi.org/10.1021/es401288x

[9]Goudriaan, M.; Hernando-Morales, V.; van der Meer, M.T.J.; Mets, A.; Ndhlovu, R.T.; van Heerwaarden, J.; Simon, S.; Heuer, V.B.; Hinrichs, K.-U.; Niemann, H. A Stable Isotope Assay with ^{13}C-Labeled Polyethylene to Investigate Plastic Mineralization Mediated by *Rhodococcus ruber*. *Mar. Pollut. Bull.* **2023**, *186*, 114369. https://doi.org/10.1016/j.marpolbul.2022.114369

[10]Vaksmaa, A.; Guerrero-Cruz, S.; Goudriaan, M.; Kienhuis, M.V.M.; Posthuma, I.; Gerritse, J.; Boekhout, T.; Niemann, H. Polyethylene Degradation and Assimilation by the Marine Yeast *Rhodotorula mucilaginosa*. *ISME Commun.* **2023**, *3*, 68. https://doi.org/10.1038/s43705-023-00267-z

[11]Tian, L.; Kolvenbach, B.; Corvini, N.; Wang, S.; Tavanaie, N.; Wang, L.; Ma, Y.; Scheu, S.; Corvini, P.F.X.; Ji, R. Mineralization of ^{14}C-Labeled Polystyrene by *Penicillium variabile* after Ozonation Pretreatment. *New Biotechnol.* **2017**, *38*, 101–105. https://doi.org/10.1016/j.nbt.2016.07.008

[19]Zumstein, M.T.; Nelson, T.F.; Baumgartner, R.; Woebker, D.; Wagner, M.; Kohler, H.-P.E.; McNeill, K.; Sander, M. Biodegradation of Synthetic Polymers in Soils: Tracking Carbon into CO_2 and Biomass. *Sci. Adv.* **2018**, *4*, eaas9024. https://doi.org/10.1126/sciadv.aas9024

[20]Yoshida, S.; Hiraga, K.; Takehana, T.; Taniguchi, I.; Yamaji, H.; Maeda, Y.; Toyohara, K.; Miyamoto, K.; Kimura, Y.; Oda, K. A Bacterium That Degrades and Assimilates Poly(Ethylene Terephthalate). *Science* **2016**, *351*, 1196–1199. https://doi.org/10.1126/science.aad6359

[29]Austin, H.P.; Allen, M.D.; Donohoe, B.S.; Rorrer, N.A.; Kearns, F.L.; Silveira, R.L.; Pollard, B.C.; Dominick, G.; Duman, R.; El Omari, K.; Mykhaylyk, V.; Wagner, A.; Michener, W.E.; Amore, A.; Skaf, M.S.; Crowley, M.F.; Thorne, A.W.; Johnson, C.W.; Woodcock, H.L.; McGeehan, J.E.; Beckham, G.T. Characterization and Engineering of a Plastic-Degrading Aromatic Polyesterase. *Proc. Natl. Acad. Sci. USA* **2018**, *115*, E4350–E4357. https://doi.org/10.1073/pnas.171880411

[30]Knott, B.C.; Erickson, E.; Allen, M.D.; Gado, J.E.; Graham, R.; Kearns, F.L.; Pardo, I.; Topuzlu, E.; Anderson, J.J.; Austin, H.P.; Dominick, G.; Johnson, C.W.; Rorrer, N.A.; Szostkiewicz, C.J.; Copié, V.; Payne, C.M.; Woodcock, H.L.; Donohoe, B.S.; Beckham, G.T.; McGeehan, J.E. Characterization and Engineering of a Two-Enzyme System for Plastics Depolymerization. *Proc. Natl. Acad. Sci. USA* **2020**, *117*, 25476–25485. https://doi.org/10.1073/pnas.2006753117

[31]Tournier, V.; Topham, C.M.; Gilles, A.; David, B.; Folgoas, C.; Moya-Leclair, E.; Kamionka, E.; Desrousseaux, M.-L.; Texier, H.; Gavalda, S.; Cot, M.; Guémard, E.; Dalibey, M.; Nomme, J.; Cioci, G.; Barbe, S.; Chateau, M.; André, I.; Duquesne, S.; Marty, A. An Engineered PET Depolymerase to Break Down and Recycle Plastic Bottles. *Nature* **2020**, *580*, 216–219. https://doi.org/10.1038/s41586-020-2149-4

[34]Carniel, A.; Valoni, É.; Nicomedes, J.; Gomes, A.C.; de Castro, A.M. Lipase from *Candida antarctica* (CALB) and Cutinase from *Humicola insolens* Act Synergistically for PET Hydrolysis to Terephthalic Acid. *Process Biochem.* **2017**, *59*, 84–90. https://doi.org/10.1016/j.procbio.2016.07.023

[35]Lu, H.; Diaz, D.J.; Czarnecki, N.J.; Zhu, C.; Kim, W.; Shroff, R.; Acosta, D.J.; Alexander, B.R.; Cole, H.O.; Zhang, Y.; Lynd, N.A.; Ellington, A.D.; Alper, H.S. Machine Learning-Aided Engineering of Hydrolases for PET Depolymerization. *Nature* **2022**, *604*, 662–667. https://doi.org/10.1038/s41586-022-04599-z

[38]Oberbeckmann, S.; Osborn, A.M.; Duhaime, M.B. Microbes on a Bottle: Substrate, Season, and Geography Influence the Community Composition of Microbes Colonizing Marine Plastic Debris. *PLoS ONE* **2016**, *11*, e0159289. https://doi.org/10.1371/journal.pone.0159289

[40]Pinnell, L.J.; Turner, J.W. Shotgun Metagenomics Reveals the Benthic Microbial Community Response to Plastic and Bioplastic in a Coastal Marine Environment. *Front. Microbiol.* **2019**, *10*, 1252. https://doi.org/10.3389/fmicb.2019.01252

[41]Agostini, L.; Moreira, J.; Bendia, A.G.; Kmit, M., Waters, L.G.; Santana, M.; Sumida, P.; Turra, A.; Pellizari, V.H. Deep-sea plastisphere: Long-term colonization by plastic-associated bacterial and archaeal communities in the Southwest Atlantic Ocean. *Sci. Total Environ.* **2021**, *793*, 148335. https://doi.org/10.1016/j.scitotenv. 2021.148335

[42]Lobelle, D.; Cunliffe, M. Early Microbial Biofilm Formation on Marine Plastic Debris. *Mar. Pollut. Bull.* **2011**, *62*, 197–200. https://doi.org/10.1016/j.marpolbul.2010.10.013

[43]Paço, A.; Duarte, K.; da Costa, J.P.; Santos, P.S.M.; Pereira, R.; Pereira, M.E.; Freitas, A.C.; Duarte, A.C.; Rocha-Santos, T.A.P. Biodegradation of Polyethylene Microplastics by the Marine Fungus *Zalerion maritimum*. *Sci. Total Environ.* **2017**, *586*, 10–15. https://doi.org/10.1016/j.scitotenv.2017.02.017

[44]Park, S.Y.; Kim, C.G. Biodegradation of Micro-Polyethylene Particles by Bacterial Colonization of a Mixed Microbial Consortium Isolated from a Landfill Site. *Chemosphere* **2019**, *222*, 527–533. https://doi.org/10.1016/j.chemosphere.2019.01.159

[45]Zhang, A.; Hou, Y.; Wang, Q.; Wang, Y. Characteristics and Polyethylene Biodegradation Activity of a Novel Cold-Adapted Bacterial Laccase from the Antarctic Sea-Ice Psychrophile *Psychrobacter* sp. NJ228. *J. Hazard. Mater.* **2022**, *439*, 129656. https://doi.org/10.1016/j.jhazmat.2022.129656

[46]Zhang, A.; Hou, Y.; Wang, Y.; Wang, Q.; Shan, X.; Liu, J. Highly Efficient Low-Temperature Biodegradation of Polyethylene Microplastics Using a Cold-Active Laccase Cell-Surface Display System. *Bioresour. Technol.* **2023**, *382*, 129164. https://doi.org/10.1016/j.biortech.2023.129164

[47]Kang, B.R.; Kim, S.B.; Song, H.A.; Lee, T.K. Accelerating the Biodegradation of High-Density Polyethylene (HDPE) Using *Bjerkandera adusta* TBB-03 and Lignocellulosic Substrates. *Microorganisms* **2019**, *7*, 304. https://doi.org/10.3390/microorganisms7090304

[48]Safdar, A.; Ismail, F.; Imran, M. Biodegradation of Synthetic Plastics by the Extracellular Lipase of *Aspergillus niger*. *Environ. Adv.* **2024a**, *17*, 100563. https://doi.org/10.1016/j.envadv.2024.100563

[49]Sowmya, H.V.; Ramalingappa, B.; Nayanashree, G.; Thippeswamy, B.; Krishnappa, M. Polyethylene Degradation by Fungal Consortium. *Int. J. Environ. Res.* **2015**, *9*, 823–830

[50]Alidoosti, F.; Giyahchi, M.; Moghimi, H. Synergistic Bioremediation: Fungal–Bacterial Partnership Degrades LDPE Microplastics Twice as Fast. *Curr. Res. Microb. Sci.* **2025**, *9*, 100450. https://doi.org/10.1016/j.crmicr.2025.100450

[51]Bai, F.; Fan, J.; Zhang, X.; Wang, X.; Liu, S. Biodegradation of Polyethylene with Polyethylene-Group-Degrading Enzyme Delivered by the Engineered *Bacillus velezensis*. *J. Hazard. Mater.* **2025**, *488*, 137330. https://doi.org/10.1016/j.jhazmat.2025.137330

[52]Auta, H.S.; Abioye, O.P.; Aransiola, S.A.; Bala, J.D.; Chukwuemeka, V.I.; Hassan, A.; Aziz, A.; Fauziah, S.H. Enhanced Microbial Degradation of PET and PS Microplastics under Natural Conditions in Mangrove Environment. *J. Environ. Manag.* **2022**, *304*, 114273. https://doi.org/10.1016/j.jenvman.2021.114273

[53]Gambarini, V.; Pavlov, N.; Young, P.; Dawes, S.; Auffret, A.; Kingsbury, J.M.; Donaldson, L.A.; Smith, D.A.; Weaver, L.; Pantos, O.; Handley, K.M.; Lear, G. Molecular Mechanisms of Plastic Biodegradation by the Fungus *Clonostachys rosea*. *mBio* **2025**, *16*, e00335-25. https://doi.org/10.1128/mbio.00335-25

[54]Maisto, M.; Zuzolo, D.; Tartaglia, M.; Prigioniero, A.; Ranauda, M.A.; Germinario, C.; Falzarano, A.; Castelvetro, V.; Sciarrillo, R.; Guarino, C. Advances in Plastic Mycoremediation: Focus on the Isoenzymes of the Lignin-Degradation Complex. *Sci. Total Environ.* **2024**, *948*, 174554. https://doi.org/10.1016/j.scitotenv.2024.174554

[55]Nawar, A.M.; Mostafa, A.M.; El-Kassas, S.; Rashwan, A.G.; Abdelnasser, E.; Assas, M. Protein-Mediated Microbial Biodegradation of High-Density Polyethylene: A Comparative Study of *Bacillus cereus* and *Bacillus subtilis*. *Open Vet. J.* **2025**, *15*, 5616–5626. https://doi.org/10.5455/OVJ.2025.v15.i11.18

[56]Wang, L.; Huang, J.; Xu, J.; Li, X.; Ma, H.; Qian, X.; Abolfathi, S. Comparative Analysis of Microbial Colonization and Degradation of Low-Density (LDPE) and High-Density Polyethylene (HDPE) Microplastics. *Process Saf. Environ. Prot.* **2025b**, *203*, 107956. https://doi.org/10.1016/j.psep.2025.107956

[57]Afify, M.G.E.; Gomaa, O.M.; El Kareem, H.A. et al. Promoting bacterial colonization and biofilm formation for enhanced biodegradation of low-density polyethylene microplastics. *Bioresour. Bioprocess*. **2025**, *12*, 59 https://doi.org/10.1186/s40643-025-00902-8

[58]Fang, X.; Cai, Z.; Wang, X.; Liu, Z.; Lin, Y.; Li, M.; Gong, H.; Yan, M. Isolation and Identification of Four Bacterial Strains with Potential to Biodegrade Polyethylene and Polypropylene from Mangrove Environments. *Microorganisms* **2024**, *12*, 2005. https://doi.org/10.3390/microorganisms12102005

[59]Joshi, G.; Goswami, P.; Jha, D.K.; Vinithkumar, N.V.; Dharani, G. Microplastics under Siege: Biofilm-Forming Marine Bacteria from the Microplastisphere and Their Role in Plastic Degradation. *Sci. Total Environ.* **2025**, *980*, 179539. https://doi.org/10.1016/j.scitotenv.2025.179539

[60]Vidal-Verdú, À.; Latorre-Pérez, A.; Molina-Menor, E.; Baixeras, J.; Peretó, J.; Porcar, M. Living in a Bottle: Bacteria from Sediment-Associated Mediterranean Waste and Their Potential Growth on Polyethylene Terephthalate. *Microbiology Open* **2022**, *11*, e1259. https://doi.org/10.1002/mbo3.1259

[61]Hu, Q.; Jayasinghe-Arachchige, V.M.; Prabhakar, R. Degradation of the Major Plastic Pollutant Polyethylene Terephthalate by Two Distinct Proteases (Neprilysin and a Cutinase-Like Enzyme). *J. Chem. Inf. Model.* **2021**, *61*, 764–776.

[62]Vertommen, M.A.M.E.; Nierstrasz, V.A.; van der Veer, M.; Warmoeskerken, M.M.C.G. Enzymatic Surface Modification of Poly(Ethylene Terephthalate). *J. Biotechnol.* **2005**, *120*, 376–386. https://doi.org/ 10.1016/j.jbiotec.2005.06.015

[63]Duan, Y.; Yin, Y.; Ni, Z.; Liu, J.; Gui, H.; Wu, D.; Wu, X.; Wang, L. The Effective and Green Biodegradation of Polyethylene Microplastics by Screening a Bacterial Strain and Its Degrading Enzymes. *Biochem. Eng. J.* **2024**, *210*, 109429. https://doi.org/10.1016/j.bej.2024.109429

[64]Safdar, A.; Ismail, F.; Iftikhar, H.; Majid Khokhar, A.; Javed, A.; Imran, M.; Safdar, B. Determination of the Biodegradation Potential of *Aspergillus niger*, *Candida albicans*, and *Acremonium sclerotigenum* toward Polyethylene, Polyethylene Terephthalate, and Polystyrene Microplastics. *Int. J. Microbiol.* **2024b**, *2024*, 7682762. https://doi.org/10.1155/2024/7682762

[65]Aguiar, A.; Gama, L.; Fornari, M.; Neto, A.; de Souza, R.; Perna, R.; et al. Bioprospecting of Mangrove Filamentous Fungi for the Biodegradation of Polyethylene Microplastics. *J. Mar. Sci. Eng.* **2024**, *12*, 1629. https://doi.org/10.3390/jmse12091629

[66]Annenkov, V.V.; Pal'shin, V.A.; Danilovtseva, E.N.; Polyakova, M.S.; Mincheva, E.V. Nanoplastics as a Substrate for Fungal Growth *Lecanicillium coprophilum* (Cordycipitaceae, Hypocreales). *Limnol. Freshw. Biol.* **2025**, *6*, 1329–1342. https://doi.org/10.31951/2658-3518-2025-A-6-1329

[67]Arshad, I.; Sajid, S.; Yu, Q.; Li, J.; Zveushe, O.K.; Rehman, S.; Lal, M.M.; Atakpa, E.O.; Chen, X.; Zhang, W.; Li, Z.; Lei, Z.; Zhang, B.; Dong, F.; Han, Y. Elucidating Polyethylene and Polypropylene Microplastics Degradation Mechanisms and Metabolic Pathways via Pectin-Biochar-Mediated Fungal Consortia. *J. Hazard. Mater.* **2025**, *496*, 139217. https://doi.org/10.1016/j.jhazmat.2025.139217

[68]Asees, W.K.; Divya, B. A Comprehensive Approach to Evaluate Microplastic Biodegradation Potential of Mangrove Rhizobacteria. *J. Environ. Biol.* **2024**, *45*, 317–329. https://doi.org/ 10.22438/jeb/45/3/MRN-5185

[69]Pathak, V.M.; Navneet. Exploitation of Bacterial Strains for Microplastics (LDPE) Biodegradation. *Chemosphere* **2023**, *316*, 137845. https://doi.org/10.1016/j.chemosphere.2023.137845

[70]Cecilia, O.K.; Asitok, A.D.; Edet, U.O.; Ekpenyong, M.; Antai, S.P. Biodegradation of Sachet Water Polyethylene Plastic Waste Using Bacterial Isolates. *Antonie Van Leeuwenhoek* **2025**, *118*, 197. https://doi.org/10.1007/s10482-025-02202-9

[71]Ferreira-Filipe, D.A.; Oliveira, L.; Paço, A.; Fernandes, A.J.S.; Costa, F.M.; Duarte, A.C.; Rocha-Santos, T.; Patrício Silva, A.L. Biodegradation of E-Waste Microplastics by *Penicillium*

brevicompactum. *Sci. Total Environ.* **2024**, *935*, 173334. https://doi.org/10.1016/j.scitotenv.2024.173334

[72]Giyahchi, M.; Moghimi, H. Aerobic Biodegradation of Untreated Polyester–Polyether Urethanes by Newly Isolated Yeast Strains *Exophiala* sp. NS-7 and *Rhodotorula* sp. NS-12. *Sci. Rep.* **2023**, *13*, 5016. https://doi.org/10.1038/s41598-023-31639-z

[73]Jeyavani, J.; Al-Ansari, M.M.; Aldahmash, N.; Govindarajan, M.; Nicoletti, M.; Vaseeharan, B. Polypropylene microplastics mitigation by formulated bacterial consortia: Sustainable and eco-friendly approach. *J. Environ. Chem. Eng.* **2025**, *13*(6), 120325. https://doi.org/10.1016/j.jece.2025.120325

[74]Khalil, K.; Mohamad Desa, A.L.; Ruslan, N.A.; Mohd Yasin, N.H.; Anuar, F.H.; Aqma, W.S. Evaluation of *Bacillus*-Associated Polyethylene Terephthalate (PET) Surfaces for Biodegradation. *Malays. Appl. Biol.* **2025**, *54*, 19–27. https://doi.org/10.55230/mabjournal.v54i2.3197

[75]Pereira, S.; Rubina, M.; Roga, A.; Selga, T.; Skinderskis, E.; Gudrā, D.; Kalniņa, I.; Vonda, K.; Fridmanis, D.; Muter, O. Evaluation of Functional Capacity and Plastic-Degrading Potential of *Bacillus* spp. and Other Bacteria Derived from the Getliņi Landfill (Latvia). *Environ. Res.* **2025**, *279*, 121849. https://doi.org/10.1016/j.envres.2025.121849

[76]Saboor, A.; Ghufran, M.A.; Bangash, N.; Durrani, H.; Shakeel, M.T. Microplastics in Coastal Sediments of Pakistan: Site-Specific Patterns and Biodegradation by Native Bacterial Isolates. *J. Ecol. Eng.* **2026**, *27*, 69–80. https://doi.org/10.12911/22998993/209092

[77]Shaikh, S.S.; Pathade, G.R. Biodegradation of Polyethylene Using *Lysinibacillus macroides*: Isolation, Characterization, and Evaluation. *Nat. Environ. Pollut. Technol.* **2025**, *24*, B4276. https://doi.org/10.46488/NEPT.2025.v24i03.B4276

[78]Stefanović, S.; Dimitrijević, M.; Mutavdžić, D.; Atlagić, K.; Krnjajić, S.; Marjanović, Ž. *Tenebrio molitor* as an Efficient Pretreatment Bioagent for Initial Polystyrene Deterioration and Subsequent Biodegradation by *Pleurotus eryngii* and *Trametes versicolor*. *Polymers* **2025**, *17*, 1772. https://doi.org/10.3390/polym17131772

[79]Tiwari, N.; Santhiya, D.; Sharma, J.G. Significance of Landfill Microbial Communities in the Biodegradation of Polyethylene and Nylon 6,6 Microplastics. *J. Hazard. Mater.* **2024**, *462*, 132786. https://doi.org/10.1016/j.jhazmat.2023.132786

[80]Wang, Z.; Li, Y.; Bai, L.; Hou, C.; Zheng, C.; Li, W. Biodegradation of Polypropylene Microplastics by *Bacillus pasteurii* Isolated from Gold Mine Tailings. *Emerg. Contam.* **2025a**, *11*, 100397. https://doi.org/10.1016/j.emcon.2024.100397

[81]Wichatham, K.; Piyaviriyakul, P.; Boontanon, N.; Surinkul, N.; Visvanathan, C.; Fujii, S.; Boontanon, S.K. Biodegradation of Polypropylene Plastics *In Vitro* and under Natural Conditions by *Streptomyces* sp. Isolated from Plastic-Contaminated Sites. *Environ. Technol. Innov.* **2024**, *35*, 103681. https://doi.org/10.1016/j.eti.2024.103681

[82]Ameen, F.; Al-Shwaiman, H.A.; Almalki, R.; et al. Degradation of Polyvinyl Chloride (PVC) Microplastics Employing the Actinobacterial Strain *Streptomyces gobitricini*. *Biodegradation* **2025**, *36*, 19. https://doi.org/10.1007/s10532-025-10115-7

[83]Bule Možar, K.; Miloloža, M.; Martinjak, V.; Ujević Bošnjak, M.; Markić, M.; Bolanča, T.; Cvetnić, M.; Kučić Grgić, D.; Ukić, Š. The Potential of AOP Pretreatment in the Biodegradation of PS and PVC Microplastics by *Candida parapsilosis*. *Water* **2024**, *16*, 1389. https://doi.org/10.3390/w16101389

[84]Carvalho, L.; Lopes, I.; Magalhães, S.; Cardoso, P.; Venâncio, C. Comparative Biodegradation of Polyethylene Terephthalate by Two White-Rot Fungi: *Trametes versicolor* and *Pleurotus sajor-caju*. *J. Clean. Prod.* **2025**, *533*, 146892. https://doi.org/10.1016/j.jclepro.2025.146892

[85]Spina, F.; Tummino, M.L.; Poli, A.; Prigione, V.; Ilieva, V.; Cocconcelli, P.; Puglisi, E.; Bracco, P.; Zanetti, M.; Varese, G.C. Low-Density Polyethylene Degradation by Filamentous Fungi. *Environ. Pollut.* **2021**, *274*, 116548. https://doi.org/10.1016/j.envpol.2021.116548

[86]Devi, D.; Gupta, K.K.; Chandra, H.; Sharma, K.K.; Sagar, K.; Mori, E.; de Farias, P.A.M.; Coutinho, H.D.M.; Mishra, A.P. Biodegradation of Low-Density Polyethylene (LDPE) through Application of the Indigenous Strain *Alcaligenes faecalis* ISJ128. *Environ. Geochem. Health* **2023**, *45*, 9391–9409. https://doi.org/10.1007/s10653-023-01590-z

[87]Dhaka, V.; Singh, S.; Ramamurthy, P.C.; Samuel, J.; Naik, T.S.S.K.; Khasnabis, S.; Prasad, R.; Singh, J. Biological Degradation of Polyethylene Terephthalate by Rhizobacteria. *Environ. Sci. Pollut. Res.* **2022**. https://doi.org/10.1007/s11356-022-20324-9

[88]Jayan, N.; Skariyachan, S.; Sebastian, D. The Escalated Potential of the Novel Isolate *Bacillus cereus* NJD1 for Effective Biodegradation of LDPE Films without Pretreatment. *J. Hazard. Mater.* **2023**, *455*, 131623. https://doi.org/10.1016/j.jhazmat.2023.131623

[89]Lv, S.; Wang, Q.; Li, Y.; Gu, L., Hu, R.; Chen, Z.; Shao, Z. Biodegradation of Polystyrene and Polypropylene by Deep-Sea Psychrophilic Bacteria Accompanied by the Simultaneous Release of Microplastics and Nanoplastics. *Sci. Total Environ.* **2024**, *948*, 174857. https://doi.org/10.1016/j.scitotenv.2024.174857

[90]Maheswaran, B.; Al-Ansari, M.; Al-Humaid, L.; Sebastin Raj, J.; Kim, W.; Karmegam, N.; Mohamed Rafi, K. *In Vivo* Degradation of Polyethylene Terephthalate Using Microbial Isolates from Plastic-Polluted Environments. *Chemosphere* **2023**, *310*, 136757. https://doi.org/10.1016/j.chemosphere.2022.136757

[91]Mallisetty, R.; Veluru, S.; Hamzah, H.; Hamem, M.; Tukarambai, M.; Poiba, V.; et al. Biodegradation of Low-Density Polyethylene (LDPE) by *Paenibacillus* sp. and *Serratia* sp. Isolated from Marine Soil Samples. *Mater. Today Proc.* **2023**. https://doi.org/10.1016/j.matpr.2023.05.639

[92]Mishra, S.; Dash, D.; Das, A.P. Microbial Degradation of Polyester Microfibers Using an Indigenously Isolated *Exiguobacterium* sp. *CLEAN Soil Air Water* **2024**, *52*, e202300343. https://doi.org/10.1002/clen.202300343

[93]Newrick, B.A.; Valdés, D.; Laca, A.; Díaz, M. Enhanced Biodegradation of High-Density Polyethylene Microplastics: Bacterial Efficiency and Process Optimization. *J. Hazard. Mater.* **2025**, *485*, 136822. https://doi.org/10.1016/j.jhazmat.2024.136822

[94]Pramanik, A.; Prasad, R.; Singh, A.; Rasulov, A.; Azizjanov, K.; Yaxshimuratov, M.; Ataullaev, Z.; Yadav, P.; Jha, S.K. Degradation of Polypropylene and Polystyrene Microplastics Using Novel

Microbial Consortia. *J. Integr. Sci. Technol.* **2026**, *14*, 1514. https://doi.org/10.62110/sciencein.jist.2026.v14.1514

[95]Puliga, F.; Zuffi, V.; Baldo, D.; et al. *Cladosporium cladosporioides* (Strain Clc/1): A Candidate for Low-Density Polyethylene Degradation. *Chem. Biol. Technol. Agric.* **2023**, *10*, 50. https://doi.org/10.1186/s40538-023-00419-2

[96]Rad, M.M.; Moghimi, H.; Azin, E. Biodegradation of Thermo-Oxidatively Pretreated Low-Density Polyethylene (LDPE) and Polyvinyl Chloride (PVC) Microplastics by *Achromobacter denitrificans* Ebl13. *Mar. Pollut. Bull.* **2022**, *181*, 113830. https://doi.org/10.1016/j.marpolbul.2022.113830

[97]Shirazi, S.; Mafigholami, R.; Moghimi, H.; et al. Feasibility Study of Microplastic Biodegradation in Effluents from the South Tehran Wastewater Treatment Plant after Quantitative and Qualitative Particle Characterization. *Appl. Water Sci.* **2023**, *13*, 80. https://doi.org/10.1007/s13201-023-01875-9

[98]Wang, H.; Guan, F.; Zhu, Y.; Pan, Y.; Liu, Q.; Liu, Q.; He, W.; Gong, D.; Tian, J.; Han, D. Biofilm Formation Promotes Polyethylene Biodegradation by *Gordonia polyisoprenivorans* B251 Isolated after Long-Term Hexadecane Enrichment. *Chemosphere* **2023**, *344*, 140383. https://doi.org/10.1016/j.chemosphere.2023.140383

[99]Zhang, Y.; Cao, Y.; Chen, B.; Dong, G.; Zhao, Y.; Zhang, B. Marine Biodegradation of Plastic Films by *Alcanivorax* under Different Ambient Temperatures: Bacterial Enrichment, Morphological Alterations, and Release of Degradation Products. *Sci. Total Environ.* **2024**, *917*, 170527. https://doi.org/10.1016/j.scitotenv.2024.170527

[100]Puspitasari, N.; Tsai, S.-L.; Lee, C.-K. Fungal Hydrophobin RolA Enhances PETase Hydrolysis of Polyethylene Terephthalate. *Appl. Biochem. Biotechnol.* **2021**, *193*, 1284–1295. https://doi.org/10.1007/s12010-020-03358-y

[101]Mrigwani, A.; Thakur, B.; Guptasarma, P. Conversion of Polyethylene Terephthalate into Pure Terephthalic Acid through Synergy between a Solid-Degrading Cutinase and a Reaction Intermediate-Hydrolyzing Carboxylesterase. *Green Chem.* **2022**, *24*, 6707–6719. https://doi.org/10.1039/D2GC01965

[102]Lin, W.; Zhao, Y.; Su, T.; Wang, Z. Enzymatic Hydrolysis of Poly(butylene Adipate-co-Terephthalate) by *Fusarium solani* Cutinase. *Polym. Degrad. Stab.* **2023**, *211*, 110335. https://doi.org/10.1016/j.polymdegradstab.2023.110335

[103]Lopez-Lorenzo, X.; Hueting, D.; Bosshard, E.; Syrén, P. Degradation of PET Microplastic Particles to Monomers in Human Serum by PETase. *Faraday Discuss.* **2024**, *252*, 387–401. https://doi.org/10.1039/D4FD00014E

[104]Gao, R.; Liu, R.; Sun, C.A Marine Fungus, *Alternaria alternata* FB1, Efficiently Degrades Polyethylene. *J. Hazard. Mater.* **2022**, *431*, 128617. https://doi.org/10.1016/j.jhazmat.2022.128617

[105]Zhang, J.; Gao, D.; Li, Q.; Zhao, Y.; Li, L.; Lin, H.; Bi, Q.; Zhao, Y. Biodegradation of Polyethylene Microplastic Particles by the Fungus *Aspergillus flavus* Isolated from the Gut of the

Wax Moth *Galleria mellonella*. *Sci. Total Environ.* **2020**, *704*, 135931. https://doi.org/10.1016/j.scitotenv.2019.135931

[106]Sulaiman, S.; Yamato, S.; Kanaya, E.; Kim, J.J.; Koga, Y.; Takano, K.; Kanaya, S. Isolation of a Novel Cutinase Homolog with Poly(Ethylene Terephthalate)-Degrading Activity from Leaf-Branch Compost Using a Metagenomic Approach. *Appl. Environ. Microbiol.* **2012**, *78*, 1556–1562. https://doi.org/10.1128/AEM.06725-11

[107]Liu, G.; Yuan, H.; Chen, Y.; Mao, L.; Yang, C.; Zhang, R.; Zhang, G. Magnetic Silica-Coated Cutinase Immobilized via ELP Biomimetic Mineralization for Efficient Nano-PET Degradation. *Int. J. Biol. Macromol.* **2024**, *279*, 135414. https://doi.org/10.1016/j.ijbiomac.2024.135414

[108]Huang, Q.-S.; Chen, S.-Q.; Zhao, X.-M.; Song, L.-J.; Deng, Y.-M.; Xu, K.-W.; Yan, Z.-F.; Wu, J. Enhanced Degradation of Polyethylene Terephthalate (PET) Microplastics by an Engineered *Stenotrophomonas pavanii* in the Presence of Biofilm. *Sci. Total Environ.* **2024**, *955*, 177129. https://doi.org/10.1016/j.scitotenv.2024.177129

[109]Cao, Y.; Xiang, L.; Nikodinovic-Runic, J.; Maslak, V.; Jin, J-M.; Liang, C.; Tang, S-Y. Improving Enzymatic Degradation of Unpretreated Poly(Ethylene Terephthalate). *Chin. J. Catal.* **2025**, *71*, 375–389. https://doi.org/10.1016/S1872-2067(24)60232-9

[110]Chen, S.-Q.; Huang, Q.-S.; Li, Y.; Zhang, H.-R.; Huang, Y.; Wu, J.; Chen, S.; Yan, Z.-F. Chemotaxis-Driven *Stenotrophomonas pavanii* Surface-Aggregated FastPETase System for *In Situ* Degradation of PET Microplastics. *Chem. Eng. J.* **2025**, *522*, 167708. https://doi.org/10.1016/j.cej.2025.167708

[111]Xu, L.; An, X.; Jiang, H.; Pei, R.; Li, Z.; Wen, J.; Pi, W.; Zhang, Q. A Novel *Gordonia* sp. PS3 Isolated from the Gut of *Galleria mellonella* Larvae: Mechanisms of Polystyrene Biodegradation and Environmental Toxicological Evaluation. *J. Hazard. Mater.* **2025**, *488*, 137219. https://doi.org/10.1016/j.jhazmat.2025.137219

[112]Yang, G.; Huang, X.; Yu, G.; Tao, T.; Guo, K.; Hou, X.; Qi, C.; Gao, S.; Fang, Y. *Priestia megaterium* Jou-S14 Degrades Highly Crystalline Polyethylene Terephthalate Microplastics. *J. Mat.* **2026**, *507*, 141843. https://doi.org/10.1016/j.jhazmat.2026.141843

[113]Avinash, G.P.; Namasivayam, S.K.R.; Pattukumar, V.; et al. Microbial Biodegradation of Polyethylene Terephthalate Microplastics by an Indigenous *Candida tropicalis* Strain and Biocompatibility Evaluation of Microplastics-Degraded Metabolites in GIFT Tilapia. *3 Biotech* **2025**, *15*, 227. https://doi.org/10.1007/s13205-025-04396-1

[114]Zheng, Y.; Liu, Y.; Wang, S.; Tang, S.; Yu, X.; Chen, S.; Su, Y. Synergistic Degradation of Polyethylene Microplastics by Recombinant *Pichia pastoris* and Purified Laccase. *J. Clean. Prod.* **2025**, *534*, 147073. https://doi.org/10.1016/j.jclepro.2025.147073

[115]Chen, S.-Q.; Huang, Q-S.; Li, Y.; Wu, J.; Chen, S.; Yan, Z-F. Targeted Aggregation of PETase towards the Surface of *Stenotrophomonas pavanii* for Degradation of PET Microplastics. *J. Hazard. Mater.* **2024**, *480*, 135895. https://doi.org/10.1016/j.jhazmat.2024.135895

[116]Giyahchi, M.; Moghimi, H. Acceleration of a Yeast-Based Biodegradation Process of Polyethylene Terephthalate Microplastics by Tween 20: Efficiency, By-Product Analysis, and

Metabolic Pathway Prediction. *Environ. Pollut.* **2024**, *351*, 124106. https://doi.org/10.1016/j.envpol.2024.124106

[117]Giyahchi, M.; Moghimi, H. Sustainable Solution for Microplastic Removal: Sequential Biodegradation and Detoxification of Polyethylene Terephthalate Microplastics by Two Natural Microbial Consortia. *Ecotoxicol. Environ. Saf.* **2025**, *302*, 118738. https://doi.org/10.1016/j.ecoenv.2025.118738

[118]Li, X.; Wu, H.; Gong, J.; Li, Q.; Li, Z.; Zhang, J. Improvement of PET Microplastic Biodegradation Using a Whole-Cell Biocatalyst through Interface Activation Reinforcement. *Environ. Technol.* **2023**, *44*, 3121–3130. https://doi.org/10.1080/09593330.2022.2052359

[119]Lee, G.-J.; Jo, H.; Kwon, H.T.; Lim, S.I. Engineered *Vibrio natriegens* as a Living Biocatalyst for *In Situ* Biodegradation of Microplastics in Seawater. *Mar. Pollut. Bull.* **2026**, *225*, 119177. https://doi.org/10.1016/j.marpolbul.2025.119177

[120]Yip, A.; McArthur, O.D.; Ho, K.C.; Aucoin, M.G.; Ingalls, B.P. Degradation of Polyethylene Terephthalate (PET) Plastics by Wastewater Bacteria Engineered via Conjugation. *Microb. Biotechnol.* **2024**, *17*, e70015. https://doi.org/10.1111/1751-7915.70015

[121]Zeng, C.; Ding, F.; Zhou, J.; Dong, W.; Cui, Z.; Yan, X. Biodegradation of Poly(Ethylene Terephthalate) by *Bacillus safensis* YX8. *Int. J. Mol. Sci.* **2023**, *24*, 16434. https://doi.org/10.3390/ijms242216434

[122]Son, H.F.; Hwang, S.; Kim, Y.; Ahn, J.H.; Ko, J.K.; Gong, G.; Um, Y.; Park, J.H.; Park, H.J.; Lee, S.-M. Enzymatic Depolymerization of Polyethylene Using a Small Laccase and Its Potential for Bio-Upcycling. *J. Hazard. Mater.* **2025**, *495*, 139021. https://doi.org/10.1016/j.jhazmat.2025.139021

[123]Tiso, T.; Winter, B.; Wei, R.; Hee, J.; de Witt, J.; Wierckx, N.; Quicker, P.; Bornscheuer, U.T.; Bardow, A.; Nogales, J.; Blank, L.M. The Metabolic Potential of *Pseudomonas putida* KT2440 for High-Value Bioconversion of PET-Derived Monomers. *Metab. Eng.* **2021**, *66*, 28–42. https://doi.org/10.1016/j.ymben.2021.03.011

[124]Choonut, A.; Wongfaed, N.; Wongthong, L.; Poolpol, A.; Chaikitkaew, S.; Sittijunda, S.; Reungsang, A. Microbial Degradation of Polypropylene Microplastics and Concomitant Polyhydroxybutyrate Production: An Integrated Bioremediation Approach with Metagenomic Insights. *J. Hazard. Mater.* **2025**, *490*, 137806. https://doi.org/10.1016/j.jhazmat.2025.137806

[125]Alharbi, M.; Abd-Elhaliem, S.M.; Afifi, S.S.; Al-Shareef, A.; Mosbah, R.A.; Mansour, A.T.; Alharbi, N.K.; Bendary, M.M. Toward Sustainable Plastic Bioremediation Using Bacterial Consortia from Aquatic Environments. *Front. Bioeng. Biotechnol.* **2026**, *13*, 1709072. https://doi.org/10.3389/fbioe.2025.1709072

[126]Choonut, A.; Wongfaed, N.; Poolpol, A.; Boonlue, S.; Wantala, K.; Pinyakong, O.; Plangklang, P.; Reungsang, A. Biodegradation of Polyethylene Terephthalate Microplastics by *Paenibacillus naphthalenovorans* PETKKU2: Response Surface Optimization and Genomic Evidence for an Alternative Degradation Mechanism. *PLoS ONE* **2026**, *21*, e0341623. https://doi.org/10.1371/journal.pone.0341623

[127]Yolanda, D.M.; Anggiani, M.; Agung, M.U.K.; Anggraeni, S.R.; Afianti, N.F. Polystyrene Microplastics Degradation by a Microbial Consortium from Jakarta Bay. *Total Qual. Manag. Bus. Excell.* **2024**, *34*, e22291. https://doi.org/10.1002/tqem.22291

[128]Montazer, Z.; Habibi Najaf, M.B.; Levin, D.B. Microbial Degradation of Low-Density Polyethylene and Synthesis of Polyhydroxyalkanoate Polymers. *Can. J. Microbiol.* **2019**, *65*, 1–11. https://doi.org/10.1139/cjm-2018-0335

[129]Rohrbach, S.; Gkoutselis, G.; Mauel, A.; Telli, N.; Senker, J.; Ho, A.; Rambold, G.; Horn, M.A. Setting New Standards: Multiphasic Analysis of Microplastic Mineralization by Fungi. *Chemosphere* **2024**, *349*, 141025. https://doi.org/10.1016/j.chemosphere.2023.141025
